# Neuroprotective Effect of Hydrogen Sulfide Subchronic Treatment Against TBI-Induced Ferroptosis and Cognitive Deficits Mediated Through Wnt Signaling Pathway

**DOI:** 10.1007/s10571-023-01399-5

**Published:** 2023-08-25

**Authors:** Jie Chen, Zhennan Chen, Dongyu Yu, Yufei Yan, Xiuli Hao, Mingxia Zhang, Tong Zhu

**Affiliations:** 1 College of Forensic Medicine, Xi’an Jiaotong University Health Science Center, No.76 Yanta West Road, Xi’an, 710061 Shaanxi China; 2 Clinical Experimental Center, Xi’an Engineering Technology Research Center for Cardiovascular Active Pep-Tides, The Affiliated Xi’an International Medical Center Hospital, Northwest University, No.777 Xitai Road, Xi’an, 710100 Shaanxi China

**Keywords:** Traumatic brain injury, Ferroptosis, Hydrogen sulfide, Wnt signaling pathway, Neuronal damage, Cognitive impairments

## Abstract

Emerging evidence shows that targeting ferroptosis may be a potential therapeutic strategy for treating traumatic brain injury (TBI). Hydrogen sulfide (H_2_S) has been proven to play a neuroprotective role in TBI, but little is known about the effects of H_2_S on TBI-induced ferroptosis. In addition, it is reported that the Wnt signaling pathway can also actively regulate ferroptosis. However, whether H_2_S inhibits ferroptosis via the Wnt signaling pathway after TBI remains unclear. In this study, we first found that in addition to alleviating neuronal damage and cognitive impairments, H_2_S remarkably attenuated abnormal iron accumulation, decreased lipid peroxidation, and improved the expression of glutathione peroxidase 4, demonstrating the potent anti-ferroptosis action of H_2_S after TBI. Moreover, Wnt3a or liproxstatin-1 treatment obtained similar results, suggesting that activation of the Wnt signaling pathway can render the cells less susceptible to ferroptosis post-TBI. More importantly, XAV939, an inhibitor of the Wnt signaling pathway, almost inversed ferroptosis inactivation and reduction of neuronal loss caused by H_2_S treatment, substantiating the involvement of the Wnt signaling pathway in anti-ferroptosis effects of H_2_S. In conclusion, the Wnt signaling pathway might be the critical mechanism in realizing the anti-ferroptosis effects of H_2_S against TBI.

**Graphical Abstract:**

TBI induces ferroptosis-related changes characterized by iron overload, impaired antioxidant system, and lipid peroxidation at the chronic phase after TBI. However, NaHS subchronic treatment reduces the susceptibility to TBI-induced ferroptosis, at least partly by activating the Wnt signaling pathway.

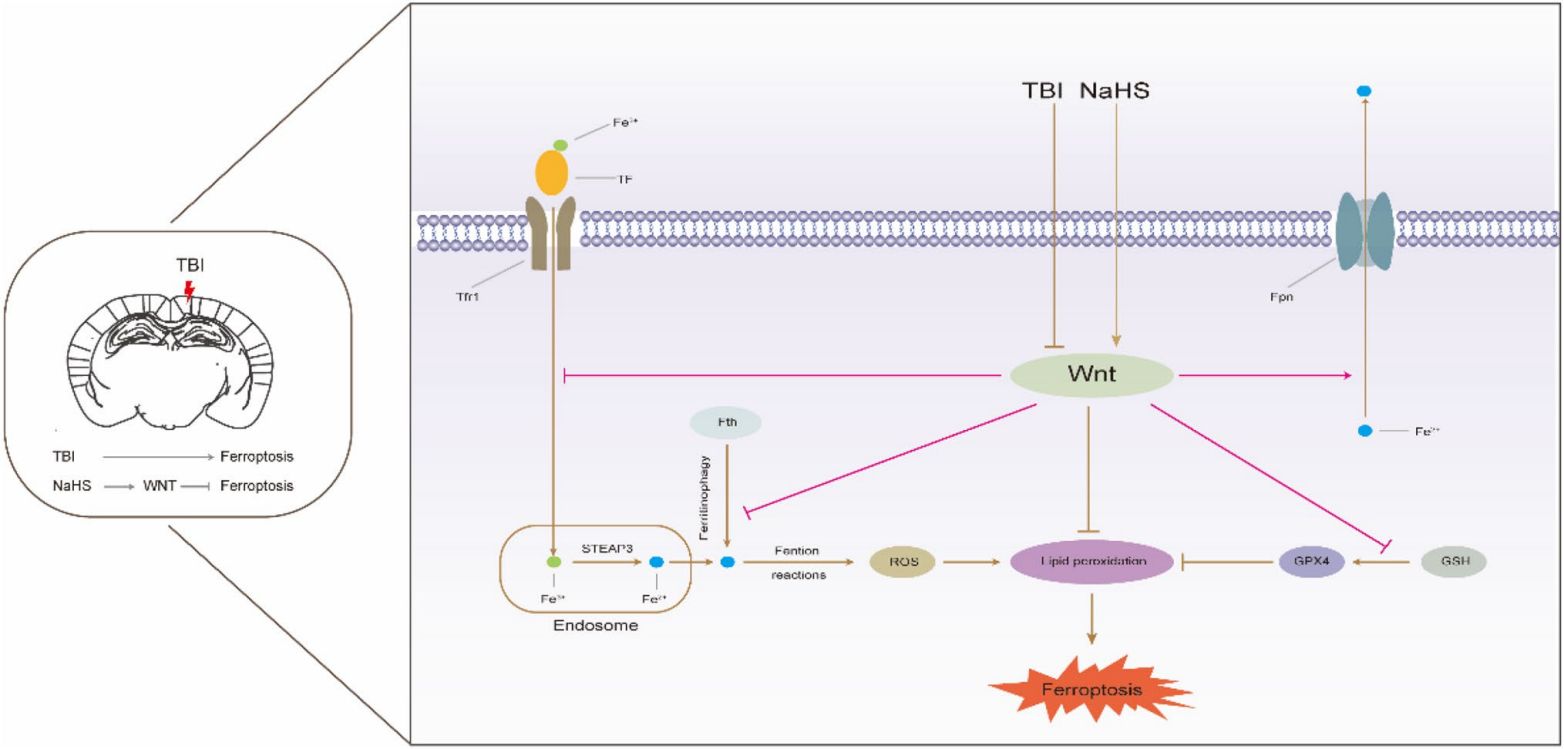

**Supplementary Information:**

The online version contains supplementary material available at 10.1007/s10571-023-01399-5.

## Introduction

Traumatic brain injury (TBI), characterized by significant morbidity and mortality, is a devastating neurological disorder worldwide, leading to tremendous social and economic expenditure each year (Meyfroidt et al. 2022). Clinical treatments have focused primarily on specialized neurointensive care, decompressive craniectomy, and pharmacological therapies (Khellaf et al. 2019). Although these therapeutic measures can reduce nerve injury to some extent, the long-term neurological and psychological outcome of patients of TBI is not optimistic. Previous studies have shown that mild TBI (mTBI) may lead to permanent memory deficits (Luo et al. 2023). In addition, TBI-induced other neurological impairments, including personality changes, behavioral disorders, and cognitive deficits, constitute significant obstacles to drug interventions and functional rehabilitation(Robert 2020). However, the specific mechanism of chronic neurocognitive impairment caused by TBI has not been fully clarified. Thus, it is urgent to develop new potential therapeutic targets and strategies to treat cognitive deficits after TBI.

Ferroptosis, a newly discovered form of regulated necrosis, is characterized by excessive iron accumulation, an impaired antioxidant system, and lethal levels of lipid peroxides (Jiang et al. 2021). It is now being appreciated that ferroptosis might be the main driving factor of neurological cell death in many neurodegenerative diseases such as Alzheimer's disease (AD) and Parkinson’s disease (PD) (Hambright et al. 2017; Mahoney-Sanchez et al. 2021). Multiple studies have established the connection between aberrant iron accumulation and cognitive decline (Bao et al. 2021). More importantly, extensive research has found that characteristics of ferroptosis, including shrunken mitochondria, abnormal iron deposition, and overproduction of free radicals, exist in the TBI mouse model, providing robust evidence for the occurrence of ferroptosis post-TBI (Geng et al. 2021). The application of several ferroptosis inhibitors (Liproxstatin-1 and Ferrostatin-1) has been found to effectively alleviate TBI-induced pathological and neurobehavioral damage, indicating that inhibiting ferroptosis may be a novel therapeutic strategy against TBI (Xie et al. 2019).

Hydrogen sulfide (H_2_S), a novel neurological gasotransmitter, has attracted intensive attention for its multiple neuroprotective functions against TBI (Huerta de la Cruz et al. 2022). For example, Chen et al. found that H_2_S could reduce the degree of neurodegeneration and promote brain repair by inhibiting neuronal pyroptosis after TBI (Chen et al. 2022). In addition, H_2_S ameliorates lipopolysaccharide (LPS)-induced behavioral deficits by facilitating microglial polarization toward M2 anti-inflammatory phenotype (Kumar et al. 2021). Furthermore, researchers have shown the protective effects of H_2_S in TBI via the reduction of intracellular calcium concentration, restoration of blood–brain barrier (BBB) integrity, and inhibition of excessive autophagy activation (Zhang et al. 2020a, b). Recently, numerous studies have revealed a close association between H_2_S and ferroptosis in other diseases. For example, Wang et al. found that H_2_S treatment reduces anxiety-like and depressive-like behaviors by inhibiting ferroptosis against type 1 diabetes mellitus (T1DM) (Wang et al. 2021). Moreover, H_2_S alleviated PM2.5 (Fine particulate matter, with less than 2.5 μm in aerodynamic diameter)-induced airway inflammation and emphysema via blocking the progression of ferroptosis, suggesting the potent anti-ferroptosis action of H_2_ S (Wang et al. 2022a, b, c, d). However, whether the anti-ferroptosis effects are involved in the neuroprotection of H_2_S after TBI is still unclear and remains to be explored.

The canonical Wnt/β-catenin pathway consists of a family of proteins necessary for embryonic development and self-renewal of adult tissues in mammals (Liu et al. 2022). Accumulating evidence suggests that the Wnt signaling pathway plays a crucial role in the pathological process of TBI (Guo et al. 2021; Li et al. 2020). Guo et al. revealed that the promotion of vascular repair and cerebrovascular remodeling is accompanied by overexpression of the Wnt signaling pathway after TBI (Guo et al. 2021). Additionally, activation of the Wnt signaling pathway contributes to neurological function recovery, possibly via its anti-inflammatory and antiapoptotic activities (Li et al. 2020). Recently, mounting evidence has revealed the close association between the Wnt signaling pathway and ferroptosis, as evidenced by the fact that activating the Wnt signaling pathway reduces cellular lipid reactive oxygen species (ROS) production and inhibits the progression of ferroptosis in gastric cancer (GC) cells (Wang et al. 2022a, b, c, d). However, whether the Wnt signaling pathway is implicated in TBI-induced ferroptosis is poorly understood. Considering that H_2_S can make cells less sensitive to ferroptosis (Li et al. 2022; Wang et al. 2022a, b, c, d; Wang et al. 2021), and the Wnt signaling pathway is the fundamental mechanism of multiple physiological effects of H_2_S in numerous pathogenetic conditions such as Diabetic cardiomyopathy (DCM) and triple-negative breast cancer (Bhattacherjee et al. 2022; Zhang and Ye 2019), it’s reasonable to propose that inhibition of ferroptosis is involved in the neuroprotection of H_2_S post-TBI, and a possible mechanism linking H_2_S to the anti-ferroptosis effects is Wnt signaling pathway.

In the present study, we first established a TBI model to investigate whether administration with H_2_S could impact ferroptosis, neuronal damage, and neurological function deficits induced by TBI. Then, Wnt3a treatment was conducted to activate the Wnt signaling pathway post-TBI, therefore exploring the effects of the Wnt signaling pathway on TBI-induced ferroptosis and neuronal loss. Finally, we performed the intraperitoneal injection of XAV939 (an inhibitor of the Wnt signaling pathway) along with H_2_S treatment post-TBI, aiming to investigate whether suppression of Wnt/β-catenin pathway could abolish the anti-ferroptosis and neuroprotective effects of H_2_S against TBI.

## Materials and Methods

### Animals

All male mice (6–8 weeks, weighting 20–25 g) were randomly housed under a controlled environment (5 animals per cage, 55 ± 5% humidity, 23 ± 1 °C, 12: 12-h light/dark cycle) and acclimatized for 7 days before use. Standard laboratory mouse diet and water were available ad libitum. All the animal experiments and procedures were approved by Xi’an Jiaotong University Laboratory Animal Administration Committee and were performed strictly in accordance with Xi’an Jiaotong University Guidelines for Animal Experimentation (No. XJ20120117) (Zhang et al. 2020a, b). Best efforts were made to minimize the number of animals used and distress to the animals.

### Traumatic Brain Injury

Controlled cortical impact (CCI) was conducted on mice to establish the TBI model using Pneumatic Impact Device (AMS 201, AmScience). In brief, mice were anaesthetized with 2% sodium pentobarbital and then mounted on a stereotactic device (RWD Life Science Co, Shenzhen, China). Under aseptic conditions, a midsagittal skin incision was made on the scalp, and a 3.0-mm diameter of circular craniotomy was then conducted over the left frontal cortex (center of the craniotomy: 2.0 mm posterior and 2.0 mm lateral to the bregma). Subsequently, the skullcap was gently removed without disruption to the dura, and the exposed cortical cortex was perpendicularly struck by a 2.0 mm-diameter flat tip with the following parameters: a depth of 1.0 mm, an impact duration time of 70 ms and a pressure of 10 kpa. After impact, the scalp was closed with tissue adhesive (3 M). After recovering for about one hour, all mice could move independently and then were returned to their respective cages. The mice in the Sham group only underwent the surgical procedure except for CCI injury (Chen et al. 2021a, b).

### Drug Administration

Sodium hydrosulfide (NaHS), an H_2_S donor, was purchased from Sigma (Sigma-Aldrich, St. Louis, MO) and dissolved in saline. NaHS (1 μmol/kg) was injected intraperitoneally daily from 1 to 7 days post-TBI. The effectiveness of NaHS dosage has been confirmed by previous studies (Zhang et al. 2014).

Liproxstatin-1 (Lip-1), a specific ferroptosis inhibitor, was obtained from TargetMol. Lip-1 was diluted by 2% dimethyl sulfoxide (DMSO), 2% Tween 80, 40% polyethylene glycol 300 (PEG 300), and double distilled water (ddH_2_O), and the final concentration of Lip-1 was 10 mg/kg. Mice in the TBI + Lip-1 group were administrated intraperitoneally with Lip-1 daily from 1 to 7 days post-TBI (Wang et al. 2022a, b, c, d).

The recombinant Wnt-3a protein (R&D Systems, Minneapolis, MN, USA) was reconstituted in saline containing 0.1% bovine serum albumin (BSA), and the final usage of Wnt-3a was 2 ng/μL. Wnt-3a was delivered intranasally at 1 day after TBI and then administered daily for 6 days. For each injection, 5 µl of Wnt-3a was delivered to each alternating nostril every 2 min until the total amount (25 μl) was delivered. The mouse was administered Wnt-3a for 50 ng once a day. The dosage and frequency of Wnt3a administration were previously described (Wei et al. 2018; Zhang et al. 2018).

XAV-939 (MCE, Monmouth Junction, NJ) was dissolved in a mixed co-solvent (10% DMSO + 90% corn oil). The final concentration of XAV-939 was 3.33 mg/ml. The mice were injected intraperitoneally with XAV-939 (40 mg/kg) at 1 day post-TBI and then administered for 6 consecutive days. The dose of XAV-939 was chosen based on previous literature (Zhou et al. 2022).

### Experimental Design

**Fig. 1:**
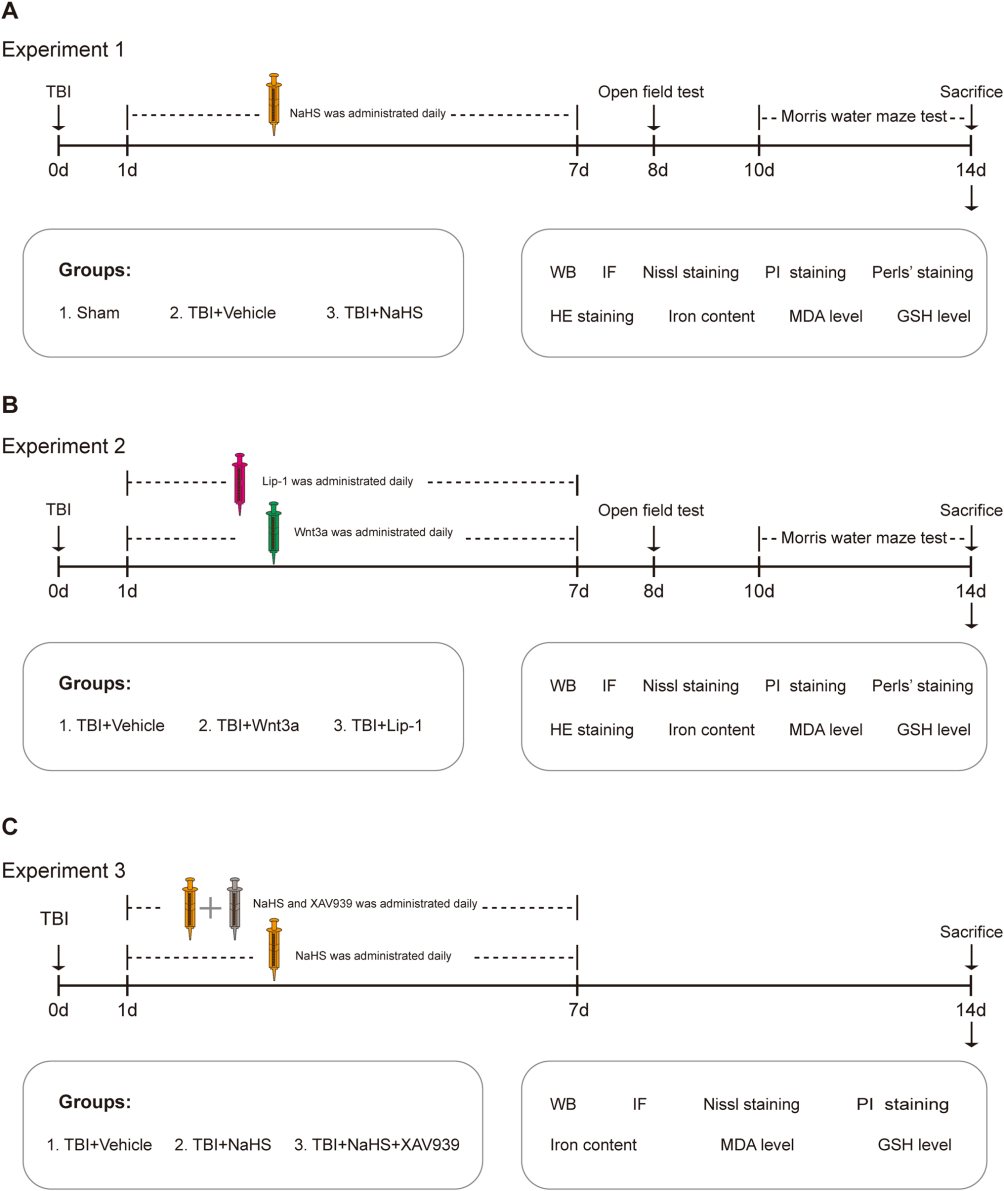
Experimental design and timeline. **A** Experiment 1 was designed to detect whether H_2_S could reduce ferroptosis and cognitive impairments caused by TBI. **B** Experiment 2 was designed to explore the effects of the Wnt signaling pathway on TBI-induced ferroptosis and neurological behavioral deficits. **C** Experiment 3 was designed to examine whether the Wnt signaling pathway was implicated in the anti-ferroptosis effects of H_2_S after TBI

Mice were randomly divided into the following groups. This study consisted of three experiment parts, and the detailed experimental design is shown in Fig. 1.

Experiment 1: To investigate the effects of H_2_S on ferroptosis and cognitive impairments induced by TBI, mice were divided into 3 groups (n = 90): Sham group (n = 30), TBI + Vehicle group (n = 30), and TBI + NaHS group (n = 30). Sham group: The mice received only the same surgical procedures, except for the CCI injury; TBI + Vehicle group: The mice were intraperitoneally injected with saline daily from 1 to 7 days after CCI injury; TBI + NaHS group: The mice were administered intraperitoneally with NaHS (1 μmol/kg) daily from 1 to 7 days post CCI injury. Then, Open field test (OFT) was performed at 8 days post-TBI, and Morris water maze (MWM) test was conducted from 10 to 14 days post-TBI. After the mice were sacrificed, Western blotting (WB), immunofluorescence (IF), Perls’ blue staining, Nissl staining, propidium iodide (PI) staining, Hematoxylin–Eosin (HE) staining, Iron content assay, malondialdehyde (MDA) content assay and reduced glutathione (GSH) content assay were conducted respectively.

Experiment 2: To better clarify the role of the Wnt signaling pathway in the pathophysiology following TBI, the mice were divided randomly into 3 groups (n = 90): TBI + Vehicle group (n = 30), TBI + Wnt3a group (n = 30), and TBI + Lip-1 group (n = 30). The mice in the TBI + Wnt3a group or TBI + Vehicle group were administrated intranasally with Wnt-3a (2 ng/μL) or vehicle control (saline containing 0.1% BSA, the volume of vehicle is the same as that of Wnt-3a solution) at 1 day post-TBI and then administered for 6 consecutive days. Mice in the TBI + Lip-1 group were administered intraperitoneally daily from 1 to 7 days post-TBI. Then, OFT was conducted at 8 days post-TBI, and the MWM test was performed from 10 to 14 days after TBI. After the mice were sacrificed, WB, IF, Perls’ blue staining, Nissl staining, PI staining, HE staining, Iron content assay, MDA content assay, and GSH content assay were performed, respectively.

Experiment 3: To examine whether the anti-ferroptosis of H_2_S against TBI is mediated via the Wnt signaling pathway, mice were divided randomly into 3 groups (n = 90): TBI + Vehicle group (n = 30), TBI + NaHS group (n = 30), and TBI + NaHS + XAV939 group (n = 30). In the TBI + Vehicle group or TBI + NaHS group, mice were intraperitoneally injected with saline or NaHS daily from 1 to 7 days post TBI; Mice in the TBI + NaHS + XAV939 group were injected intraperitoneally with XAV-939 (40 mg/kg) and NaHS daily from 1 to 7 days following TBI. Subsequently, the mice were sacrificed at 14 days post-TBI, and a series of detections (WB, IF, Nissl staining, PI staining, Iron Iron content assay, MDA content assay, and GSH content assay) were conducted, respectively.

### Western Blotting

Western blot analysis was performed according to a previous description (Rui et al. 2021). The inured ipsilateral cortical samples (the center: the impact site of the ipsilateral cortex, the range: 2 × 2 × 2 mm) were isolated and lysed in ice-cold RIPA reagent (Beyotime, Jiangsu, China) containing the phenylmethanesulfonyl fluoride (PMSF, Beyotime). After sufficient lysis, the tissue homogenates were centrifuged at 12,000 rpm for 20 min at 4 °C, and the supernatants were preserved. The protein concentration of the tissue sample was quantified using bicinchoninic acid assay (BCA) kit (Beyotime, Shanghai, China). The protein samples were separated on 8% or 10% SDS-PAGE and then transferred to polyvinylidene fluoride (PVDF) membranes (Millipore). The membranes were blocked with 5% skimmed milk for 1 h at room temperature (23 ± 1 °C) and then incubated with the following primary antibodies overnight at 4 °C: anti-Fth (1:1000, Cat# CY5648, abways, China), anti-Tfr1(1:1000, Cat# CY5396, abways, China), anti-Fpn (1:1000, Cat# 26,601–1-AP, proteintech, China), anti-Gpx4 (1:1000, Cat# CY7046, abways, China), anti-4HNE (1:1000, Cat# ab46545, abcam, USA), anti-Wnt3a (1:1000, Cat# ab219412, abcam, USA), anti-β-catenin (1:1000, Cat# 51,067–2-AP, proteintech, China) and anti-β-actin (1:1000, Cat# 66009-1-Ig, proteintech, China). On the second day, membranes were treated with the anti-rabbit secondary antibody (1:1000, Cat# sc-2004, SantaCruz, USA) or anti-mouse secondary antibody (1:1000, Cat# sc-2005, SantaCruz, USA) for 1 h at room temperature (23 ± 1 °C). The protein density was captured using the ECL chemiluminescence system (Clinx Science Instruments, China). Densitometric analysis was performed using NIH Image J software (Bethesda, MD, USA).

### Immunofluorescence Staining

Brain tissues were subjected to gradient sucrose dehydration and cut into slices of 20 μm, after the fixation with 4% paraformaldehyde (PFA) for 24 h at room temperature (23 ± 1 °C). Subsequently, brain slices were rehydrated in phosphate-buffered saline (PBS) for 10 min, then permeabilized in 0.3% TritonX-100 and 10% goat serum for 1 h. Tissue sections were cultivated overnight at 4 °C with following antibodies: anti-4 HNE (1:200, Cat# ab46545, abcam, USA), anti-Gpx4 (1:100, Cat# CY6959, abways, China), and anti-Fth (1:100, Cat# CY5648, abways, China). The sections were then incubated with Alexa Fluor 488-goat anti-rabbit (1:500, Cat# A48282TR, Invitrogen, USA) and 594-goat anti-rabbit (1:500, Cat# A-11072, Invitrogen, USA) secondary antibodies for 1 h at room temperature (23 ± 1 °C) followed by three times wash in PBS. After that, the nuclei were stained with DAPI (1:1000, Beyotime) for five minutes (Feng et al. 2022). Finally, the sections were mounted and coverslipped with Fluoromount-G. Images of the brain section stained with anti-4 HNE, anti-Gpx4, and anti-Fth were photographed by a confocal laser scanning microscope (Olympus, FV1000, Japan) and confocal software (Olympus, Fluoview Ver4.2b, Japan) under a 20 × objective lens (room temperature: 23 ± 1 °C; eye lens: 10 × ; objective lens: 20 ×) or 40 × objective lens (room temperature: 23 ± 1 °C; eye lens: 10 × ; objective lens: 40 ×). All images were captured in a dark room at 23 ± 1 °C. Each experiment was repeated at least three times to confirm the findings. The number of 4HNE-, Gpx4-, and Fth-positive cells were counted manually using NIH Image—J software (Bethesda, MD, USA) by two independent investigators blinded to the groups.

### Nissl Staining

Nissl staining was conducted to evaluate the degree of neuronal damage in the injured cortex. After washing with phosphate-buffered saline plus Tween-20 (PBST) three times, frozen brain Section (15 μm) were incubated with a Nissl staining solution (C0117, Beyotime, China) for 5 min at 37 °C. Then, sections were dehydrated in gradient ethanol and cleared in xylene before being coverslipped. The normal neurons were characterized by a round shape and high levels of Nissl body. However, other morphological abnormalities, such as condensed nuclei, shrunken vacuoles, and central chromatolysis, were observed in the injured cells (Chen et al. 2020). The images for morphological changes in cells were captured using a light microscope (Nikon, Japan), and the number of Nissl-positive neurons surrounding injury areas was counted by two independent investigators blinded to the groups using the ImageJ software.

### Perls’ Prussian Blue Staining

As described previously, Perl’s staining was performed to detect cellular iron deposition (Rui et al. 2021). After washing with PBST three times (five minutes per time), the sections were treated with Perl’s solution (5% potassium ferrocyanide/5% hydrochloric acid) for 30 min. Then, the slices were incubated in solution (0.3% hydrogen peroxide in methanol) for 15 min, which aimed to block endogenous peroxidase activity. After rising with PBST for 15 min, the sections were cultured in 3,3‐diaminobenzidine (DAB; Vector Laboratories, Burlingame, USA) for 2 min to develop the signals and then were stained with hematoxylin (Sigma‐Aldrich, St Louis, MO, USA) for counterstaining. Three sections per animal were viewed and photographed under a microscope (Nikon, Japan). Iron-positive cells were quantified by four randomly‐selected microscopic fields per image at 100× magnification surrounding the injury area. Cell quantification was performed by two independent investigators blinded to the groups. The number of cells was measured using NIH Image J software (Bethesda, MD, USA).

### Propidium Iodide Labeling

Propidium iodide (Beyotime Biotechnology, China) was diluted in 0.9% NaCI, preparing a PI solution with a 0.4 mg/mL concentration. 200 μL PI solution was administered through intraperitoneal injection at 1 h before the mouse was sacrificed (Chen et al. 2019). The brains (n = 6 per group) were quickly frozen in liquid nitrogen vapour and then cut into sections of 15 μm in cross-section. Brain sections were fixed with 100% ethanol for 10 min at room temperature (23 ± 1 °C), and at least three visual fields of the injured area under 400× magnification were randomly chosen to detect the number of PI-positive cells with a fluorescence microscope (Nikon, Japan). The PI cells were measured by two independent investigators blinded to the groups.

### Lesion Volume Assessment

Serial coronal brain tissue slices were cut at 0.5-mm intervals and stained with HE. The spared tissue volume of each section is obtained by multiplying the affected areas by 0.5 mm. The total lesion volume was calculated by adding the infarct areas of the 10 slices taken from the anterior to the posterior limits of the lesion. Data were expressed as the volume percentage of the lesion versus the contralateral hemisphere using NIH Image J software (Bethesda, MD, USA) (Xu et al. 2018). All assessments were conducted by two independent investigators blinded to the experimental groups.

### MDA Content Assay

To examine the lipid peroxide levels of the ipsilateral cortex, MDA content was measured using Lipid Peroxidation MDA Assay Kit (S0131S, Beyotime, China) according to the manufacturer’s instructions. Cortical tissues of ipsilateral hemispheres were weighted and homogenized in ice-cold PBS containing 1% phenylmethanesulfonyl fluoride (PMSF) on ice. Then tissues were centrifuged at 10,000–12,000 g for 15 min, and the supernatant was collected. After measuring the supernatant concentration with a BCA Protein Assay Kit (Thermo Scientific, NJ, USA), 0.2 ml supernatant was added to each sample or standard (0.1 mL). Then, the mixture was incubated at 100 °C for 15 min, cooled to room temperature (23 ± 1 °C) in a water bath, and centrifuged at 1000 g for 10 min at room temperature (23 ± 1 °C). Finally, 0.2 ml supernatant was added to a 96-well plate, and the absorbance was measured at 532 nm using a microplate reader. MDA content was calculated as nmol/mg (Zhang et al. 2022).

### GSH Content Assay

Total glutathione (total GS) content is composed of reduced glutathione (GSH) content and oxidized glutathione (GSSG) content, and the GSH content was calculated as Total GS-GSSG × 2. Using the GSH content Kit (S0053, Beyotime, China), the concentration of total GS and GSSG could be detected. The methods of measuring the content of total GS and GSSG were listed below:

For sample preparation, tissue was frozen in liquid nitrogen and ground into powder. Subsequently, 10 mg tissue powder was added into 30 μL protein removal reagent M solution and fully vortexed. Then 70 ul protein removal reagent M solution was added to the mixture. After incubation at 4℃ for 10 min, the mixture was centrifuged at 10,000 × g for 10 min at 4℃, and the supernatant was obtained for measurement of total GS. The absorbance of the supernatant was measured at 412 nm.

In addition, the supernatant could also be used in the measurement of GSSG after adding a GSH removal reagent. Specifically, 1/5 volume diluted GSH Removal buffer was added to the supernatant and fully vortexed (e.g., add 20 µl of diluted GSH removal buffer in 100 µl of supernatant). Then, 1/25 volume GSH removal reagent working solution was added to the mixture of supernatant and GSH Removal Buffer (e.g., add 4 μl of GSH removal reagent working solution was added to the 100 μl mixture of supernatant and GSH Removal Buffer) and mixed thoroughly. After incubating for 60 min at 25℃, the mixture was used for measuring GSSG content, and the absorbance value was examined at 412 nm. Finally, the GSH content was calculated as Total GS-GSSG × 2. GSH levels were expressed as nmol/mg (Wu et al. 2017).

### Iron Content Determination

The concentration of Ferrous ion (Fe^2+^) in the injured cortex was estimated by the iron assay kit (#ab83366, Abcam, USA) as previously described (Wang et al. 2022a, b, c, d). The detection process was conducted according to the manufacturer’s protocol. Firstly, cortical tissues were washed with cold PBS and weighed. Then, 10 mg cortical tissues were added to 100 μL iron assay buffer for sufficient homogenization. Subsequently, the homogenate was centrifuged at 16, 000 × g for 10 min, and the supernatant was left for later use. Furthermore, 50 μL supernatant of each sample was added to 96-well plates, and 5 μL assay buffer was added to each supernatant, finally replenished to 100 μL with iron assay buffer. After incubating at 37 °C for 30 min, 100 μL of iron probe was added per well and incubated at 37 °C for 1 h. Finally, the absorbance was measured using a colorimetric microplate reader at 593 nm. Fe^2+^ level was expressed as nmol/mg.

### Open Field Test

To examine the anxious behaviors, OFT was performed at 8 days after TBI. The OFT device included a black square bottom (50 × 50 cm) and a black wall (50 cm), and the apparatus was divided into the central area (25 × 25 cm) and the marginal area. Mice were placed in the corner of the apparatus and habituated to the environment for 1 min. Then, the mouse was allowed freely to explore their surroundings for 5 min. The box was cleaned with a paper towel saturated in 50% ethanol and dried thoroughly after each test session. The motion trail was videotaped with an infrared camera fixed over the box. The data, including total distance, frequency, and time in the center, were counted and analyzed using Ethovision 5.0 tracking software (Noldus, Wageningen, Netherlands) (Mohammad et al. 2023).

### Morris Water Maze Test

The spatial learning and memory performance of mice was evaluated with the MWM test, as previously described (Hu et al. 2022a, b). The test device consisted of a large round pool (diameter 120 cm, depth 50 cm) filled with opaque water (23 °C ± 1 °C) to a height of 30 cm. The pool was divided into 4 quadrants with distinct visual cues hung on the wall. An invisible circular platform, 10 cm in diameter and 2 cm beneath the water surface, was located in the northeast quadrant of the pool. The experiment was divided into two consecutive phases: the acquisition phase (10–13 days post-TBI) and the spatial probe test phase (14 days post-TBI). During the acquisition phase, each mouse was trained from 10 to 13 days post-TBI, with 4 trials per day. And the trials in each session were separated by a 10 min break. For each trial, mice were positioned facing the wall in one of the four quadrants and gently released into the pool. The mice were allowed up to a maximum of 60 s to reach the hidden platform. After reaching the platform, mice were allowed to stay there for 15 s and then placed in a dry cage until the beginning of the next trial. If mice failed to find it, all of them were guided to the submerged platform and rested for 15 s. On the 14 days after TBI, the spatial probe test was conducted. During the spatial probe test, each mouse was placed in the southwest quadrant and explored freely for 60 s without the hidden platform. All data, including frequency of crossing the platform location, latency to reach the platform and swimming distance, were automatically videotaped and analyzed with a video camera connected to a computer equipped with the Ethovision 5.0 tracking software (Noldus, Netherlands).

### Statistical Analysis

Statistical analyses were conducted using GraphPad Prism 8 Software (San Diego, CA, USA). Shapiro–Wilk test was performed to check the normality. Variance homogeneity was assessed for each dataset using the Levene test. When normal distribution and homogeneity uniformity assumptions were met, data analysis was analyzed using one-way ANOVA followed by Bonferroni post hoc test, and all data were presented as mean ± standard deviation (mean ± SD). Otherwise, we performed non-parametric tests, and the data were reported as median and interquartile range. A *p*-value less than 0.05 was considered statistically significant (D'Amico et al. 2021). All analyses were performed and conducted by investigators blind to the experimental group treatment.

## Results

### NaHS Treatment Rescued TBI-Induced Ferroptosis at Chronic Stage

**Fig. 2:**
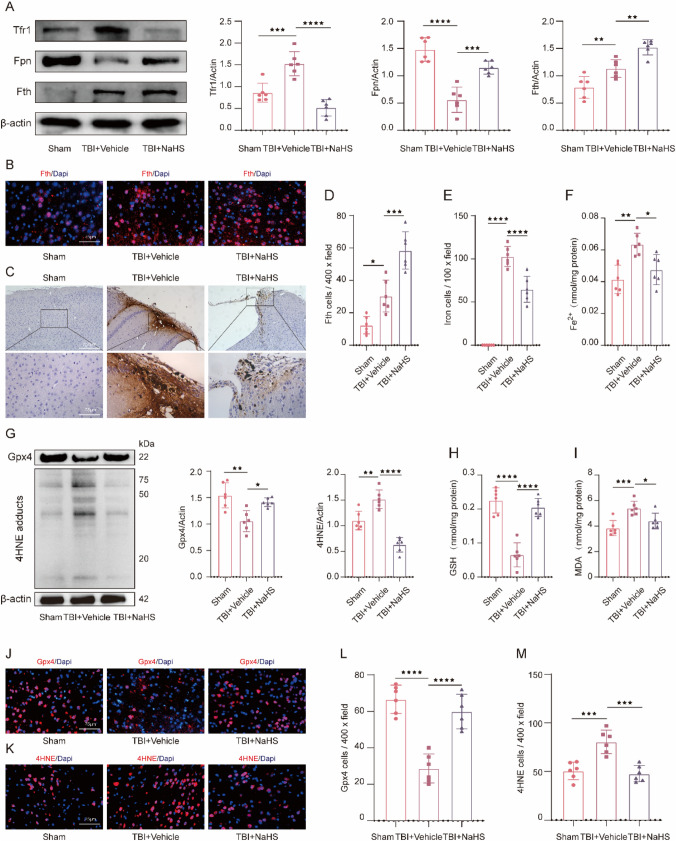
NaHS alleviated ferroptosis at the chronic phase post-TBI. **A** Representative gel bands and quantification of Tfr1 (F(2,15) = 29.57, *p* < 0.0001), Fpn (F(2,15) = 34.23, *p* < 0.0001), and Fth (F(2,15) = 27.91, *p* < 0.0001) in the injured cortex obtained from Sham, TBI + Vehicle and TBI + NaHS group at 14 days post TBI. β-actin was used as a loading control. **B** Representative images of immunofluorescent staining for Fth (red) and Dapi (blue) from each group. Magnification is 400×. Scale bar is 75 μm. **C** Representative pictures of Perls’ blue-stained cortex sections from the above groups. Magnification is 100/400×. Scale bar is 75/250 μm. **D** Quantification of Fth-positive cells per vision field (F(2,15) = 21.08, *p* < 0.0001). **E** Quantitative analysis of iron-positive cells per vision field (F(2,15) = 134.0, *p* < 0.0001). **F** The change of Fe^2+^ content (F(2,15) = 10.84, *p* < 0.0012) in the injured cortex from each group. **G** Representative immunoblot and quantification of Gpx4 (F(2,15) = 11.16, *p* = 0.0011) and 4HNE (F(2,15) = 41.38, *p* < 0.0001) in the damaged cortex from above groups. β-actin was used as a loading control. **H** The change of GSH content (F(2,15) = 40.84, *p* < 0.0001) from each group. **I** The change of MDA content (F(2,15) = 11.04, *p* = 0.0011) from each group. (J, K) Representative confocal images of Gpx4 (**J**) and 4HNE **K** staining in the injured cortex from the above groups. Magnification is 400×. Scale bar is 75 μm. **L** Quantification of Gpx4-positive cells (F(2,15) = 23.39, *p* < 0.0001) per vision field. **M** Quantification of 4HNE-positive cells (F(2,15) = 21.96, *p* < 0.0001) per vision field. All data are presented as mean ± SD (*n* = 6) and analyzed using one-way ANOVA with Bonferroni post hoc test. For all panels, *p < 0.05, **p < 0.01, ***p < 0.001, and ****p < 0.0001. All data are representative of three independent experiments

To determine the changes in iron metabolism at the chronic stage post-TBI, Western blotting was conducted to detect the expression of iron-associated proteins, including Tfr1, Fpn, and Fth. As shown in Fig. 2A, TBI led to a remarkable increase of Tfr1 (t(10) = 4.991, *p* ˂ 0.0001) and a significant decrease of Fpn (t(10) = 8.169, *p* ˂ 0.0001) compared with Sham group, and NaHS treatment effectively inversed the changes above induced by TBI (Fig. 2A). In addition, we also found that the expression of Fth was observed to increase in the TBI + Vehicle group compared to Sham group (t(10) = 3.501, *p* = 0.0097), and NaHS significantly increased the TBI-induced overexpression of Fth (t(10) = 3.966, *p* = 0.0037) relative to TBI + Vehicle group (Fig. 2A). Consistent with our Western blotting results, immunofluorescence staining results also showed that NaHS effectively increased the TBI-induced overexpression of Fth (t(10) = 3.415, *p* = 0.0115) (Fig. 2B and D). Then, Perl’s blue staining was performed to evaluate the effects of NaHS treatment on TBI-induced iron deposition, and results showed that the number of iron-positive cells was increased in the TBI + Vehicle group relative to the Sham group (t(10) = 16.19, *p* ˂ 0.0001), whereas the NaHS treatment significantly reduced the number of iron-positive cells (t(10) = 5.982, *p* ˂ 0.0001) (Fig. 2C and E). Moreover, we also found that the expression of Fe^2+^ in the TBI + NaHS group was significantly lower than that in the TBI + Vehicle group (t(10) = 3.264, *p* = 0.0157) (Fig. 2F). The above results demonstrated that iron homeostasis was impaired in the injured cortex post TBI, and NaHS treatment could reduce TBI-induced iron accumulation.

To further detect the effects of NaHS treatment on lipid peroxide and antioxidant system, Western blotting for Gpx4 and 4HNE was conducted. As depicted in Fig. 2G, TBI led to a significant increase of 4HNE (t(10) = 4.259, *p* = 0.0021) and a remarkable decrease of Gpx4 (t(10) = 4.560, *p* = 0.0011), compared with Sham group. As expected, the above changes were inverted by NaHS treatment (Fig. 2G). As the co-factor of glutathione Gpx4, GSH was considered the negative regulator of ferroptosis. The results revealed that TBI effectively reduced the expression of GSH (t(10) = 8.301, *p* ˂ 0.0001) compared to the Sham group, and NaHS significantly reversed the decrease of GSH induced by TBI (t(10) = 7.246, *p* ˂ 0.0001) (Fig. 2H). Given that MDA is an end-product of lipid peroxidation, we measured and found that NaHS treatment inhibited the TBI-induced overexpression of MDA (t(10) = 2.989, *p* = 0.0275) (Fig. 2I). Consistent with our Western blotting results, immunofluorescence staining results also showed that NaHS treatment inversed the upregulation of 4HNE (t(10) = 4.471, *p* = 0.0013) and downregulation of Gpx4 expression (t(10) = 6.799, *p* ˂ 0.0001) induced by TBI (Fig. 2J–M). These data demonstrated that NaHS treatment potently alleviates iron accumulation, restores Gpx4 activity, and reduces lipid peroxidation, thus ameliorating TBI-induced ferroptosis.

### NaHS Treatment Alleviated TBI-Induced Neuronal Damage

**Fig. 3:**
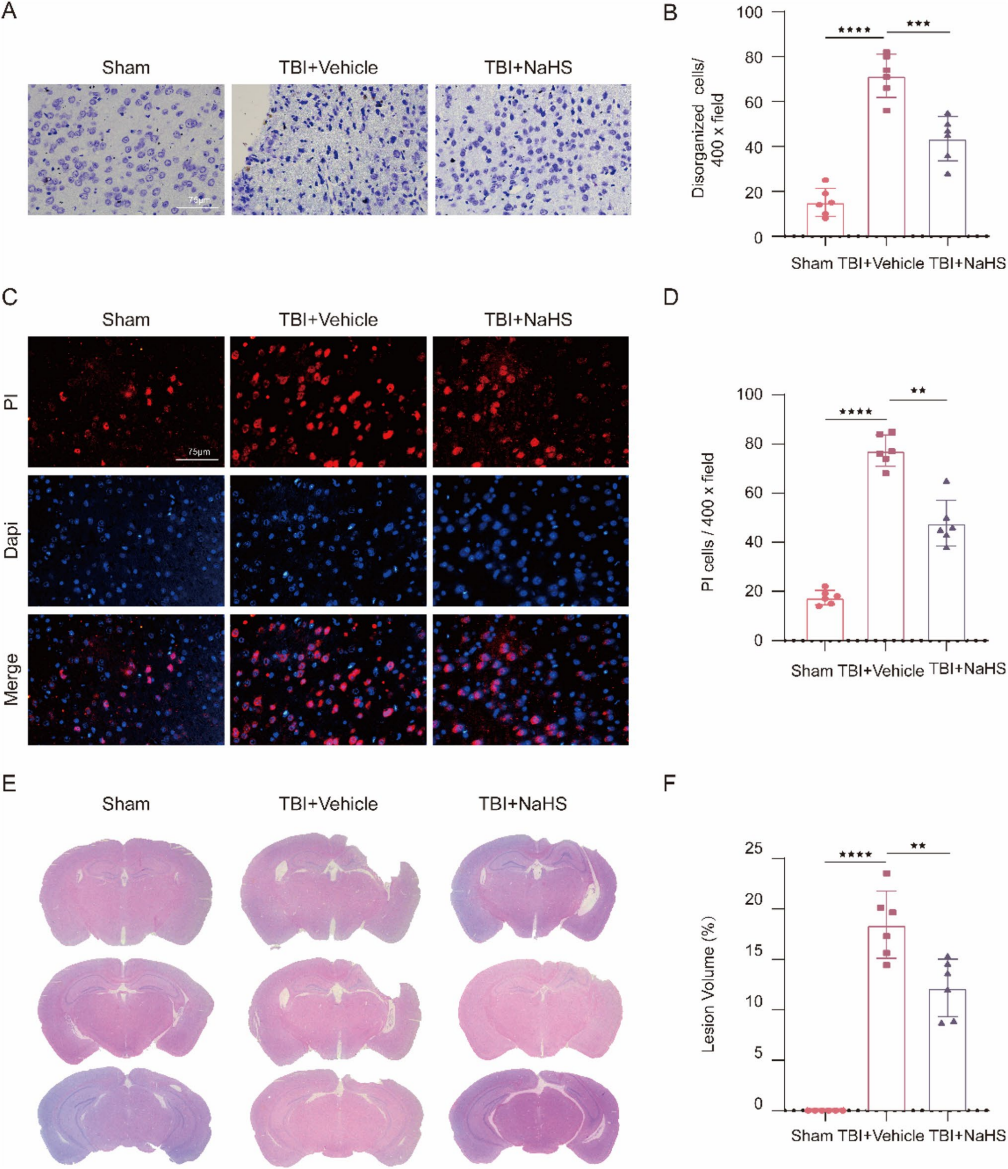
NaHS ameliorated TBI-induced neuronal damage. **A** Representative images of Nissl staining in the injured cortex from Sham, TBI + Vehicle, and TBI + NaHS group. Magnification is 400×. Scale bar is 75 μm. **B** Quantification of disorganized cells per vision field (F(2,15) = 62.86, *p* < 0.0001). **C** Representative photomicrographs of double staining with PI (red) and DAPI (blue). Magnification is 400×. Scale bar is 75 μm. **D** Quantification of PI-positive cells per vision field (F(2,15) = 119.2, *p* < 0.0001). **E** Brain sections stained with HE staining. **F** Lesion volume percentage presented as the lesion to the contralateral hemisphere (F(2,15) = 82.37, *p* < 0.0001). All data are presented as mean ± SD (*n* = 6) and analyzed using one-way ANOVA with Bonferroni post hoc test. For all panels, **p < 0.01, ***p < 0.001, and ****p < 0.0001. All data are representative of three independent experiments

To detect the changes in neuronal cell outline post-TBI, brain sections were stained with Nissl staining. The normal neurons in the Sham group were characterized by round shape, lilac-blue, relatively large soma, and high levels of Nissl body. However, the injured neurons caused by TBI showed other cell forms such as dark colour stained, condensed nuclei, shrunken vacuoles, and decreased expression of Nissl body (Fig. 3A). And, we found that the number of injured neurons was reduced in the TBI + NaHS group (t(10) = 5.573, *p* = 0.0002), compared with the TBI + Vehicle group (Fig. 3B). Moreover, PI staining was conducted to investigate the effects of NaHS treatment on cell death post-TBI, and the results showed that NaHS treatment reduced the TBI-induced increase of PI-positive cells (t(10) = 7.613, *p ˂* 0.0001) (Fig. 3C and D). In addition, we performed HE staining on the tissue sections and found that the spared hemispheric volume in the TBI + NaHS group was remarkably smaller than that in the TBI + Vehicle group (t(10) = 4.286, *p* = 0.0020) (Fig. 3E and F). Taken together, NaHS significantly could decrease the sensitivity to TBI-induced neuronal damage.

### NaHS Improved Function Outcome in Mice After TBI

**Fig. 4:**
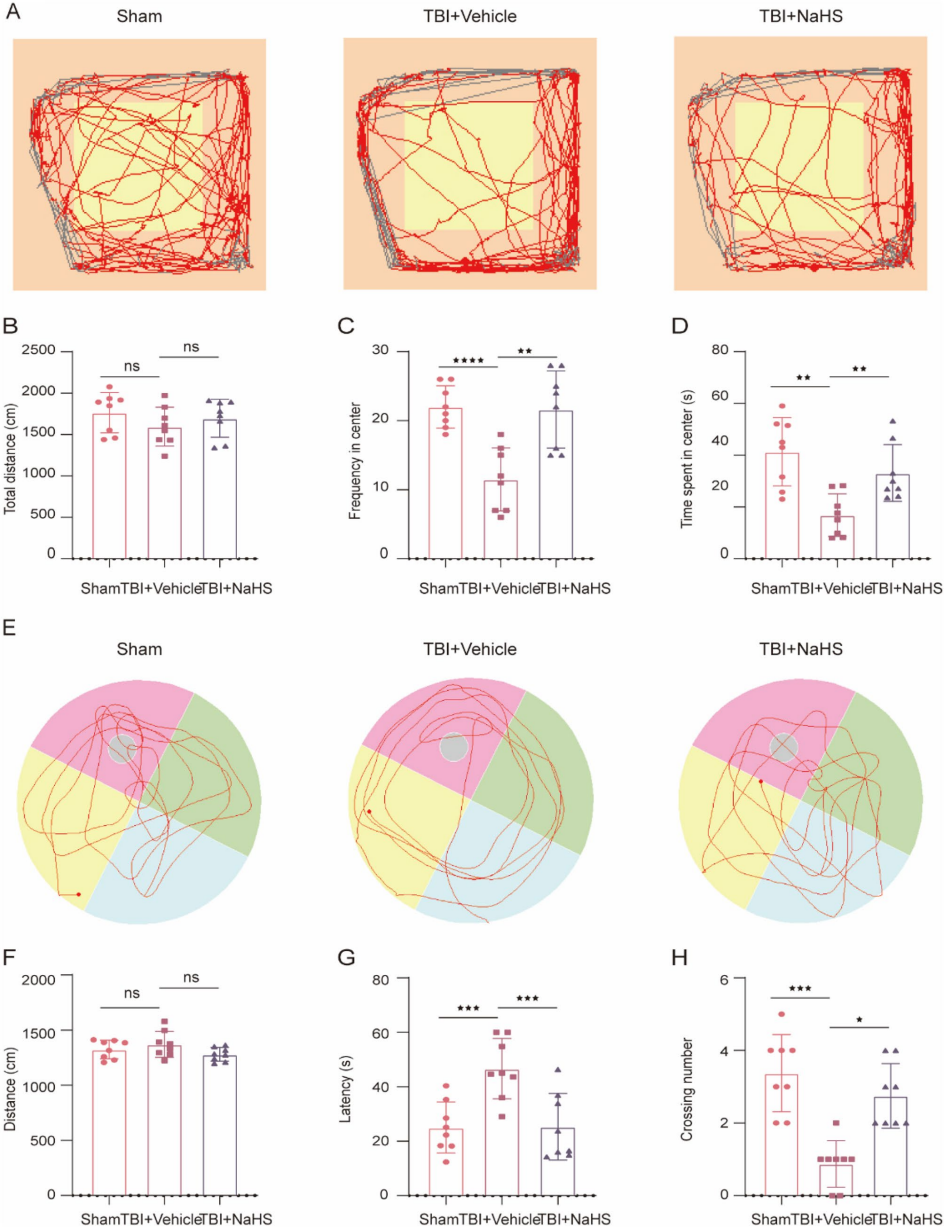
NaHS treatment reduced anxiety and spatial memory deficits induced by TBI in mice. **A**–**D** The effect of NaHS treatment on anxiety-like behaviors post-TBI. **A** Representative mouse tracking from Sham, TBI + Vehicle, and TBI + NaHS group under OFT. **B** Total distance traveled in the OFT (F(2,21) = 1.039, *p* = 0.3712). **C** Frequency into the central field by mice (F(2,21) = 13.87, *p* < 0.0001). **D** Time spent in the central field by mice (F(2,21) = 10.27, *p* = 0.0008). (E–H) The effect of NaHS treatment on mice's learning and memory function from the above groups. **E** Representative swimming path of mice from all groups in the MWM test. **F** The traveled distance during the probe trial (F(2,21) = 2.042, *p* = 0.1547). **G** Escape latency to find platform during the probe trial (F(2,21) = 17.50, *p* < 0.0001). (H) The number of crossings over the platform position during the probe trial (F(2,21) = 10.22, *p* = 0.0008). Data are shown as mean ± SD (*n* = 8 per group) and analyzed using one-way ANOVA with Bonferroni post hoc test. For all panels, *p < 0.05, **p < 0.01, ***p < 0.001, ****p < 0.0001 and ns means not statistically significant

To determine the effect of NaHS treatment on TBI-induced anxiety-like behaviors, OFT was conducted at 8 days post-TBI. The results from OFT showed no significant difference in distance covered among all groups (F(2,21) = 1.039, *p* = 0.3712) (Fig. 4B). In addition, we found that the frequency (t(14) = 4.641, *p* = 0.0004) and time spent in the center zone (t(14) = 4.455, *p* = 0.0007) in the TBI + Vehicle group were both reduced, compared to Sham group (Fig. 4A, C and D). However, NaHS treatment significantly reversed the abovementioned changes caused by TBI (Fig. 4A, C and D), demonstrating that TBI-induced anxiety-like behaviors were alleviated by NaHS sub-chronic treatment. To determine the effects of NaHS treatment on learning and memory ability, MWM test was performed from 10 to 14 days following TBI. The results revealed that no statistical difference in distance was detected from all groups (F(2,21) = 2.042, *p* = 0.1547) (Fig. 4F), indicating that swimming ability was not affected by TBI or NaHS intervention. However, the increased platform-crossing frequencies (t(14) = 4.263, *p* = 0.0010) and decreased latency time (t(14) = 3.890, *p* = 0.0025) were shown in the TBI + NaHS group (Fig. 4E, G and H), compared to TBI + Vehicle group, suggesting the recovery of spatial learning and memory function under NaHS treatment post-TBI. These results demonstrated that TBI-induced anxious-like behaviors and impaired spatial learning and memory ability might be partly alleviated by NaHS treatment post-TBI.

### Wnt3a or Lip-1 Treatment Inhibited the Sensitivity to TBI-Induced Ferroptosis

**Fig. 5:**
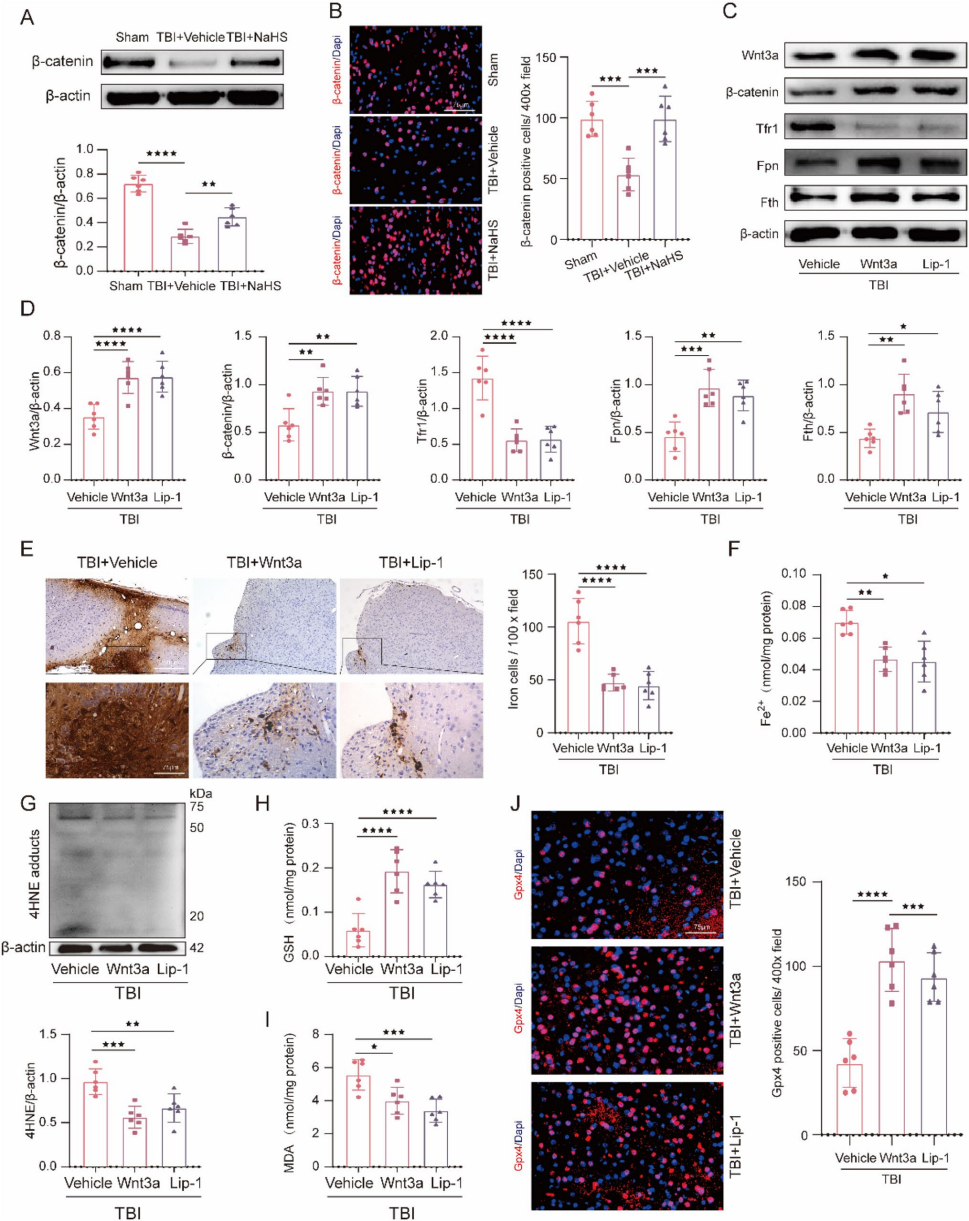
Wnt3a or Lip-1 mitigated TBI-caused ferroptosis by reducing iron homeostasis disorders, decreasing lipid peroxidation, and improving the antioxidant system. **A** Representative immunoblot and quantification of β-catenin in the injured cortex tissue from Sham, TBI + Vehicle, and TBI + NaHS group (F(2,15) = 62.94, *p* < 0.0001). β-actin was used as a loading control. **B** Representative images of immunofluorescent staining for β-catenin (red) and Dapi (blue). Magnification is 400×. Scale bar is 75 μm. Quantification of β-catenin positive cells per vision field (F(2,15) = 17.76, *p* < 0.0001). **C** Representative gel bands of Wnt3a, β-catenin, Tfr1, Fpn, Fth, and β-actin in the injured cortex from the TBI + Vehicle, TBI + Wnt3a, and TBI + Lip-1 group. **D** Quantification of Wnt3a (F(2,15) = 14.42, *p* = 0.0003), β-catenin (F(2,15) = 10.01, *p* = 0.0017), Tfr1 (F(2,15) = 29.60, *p* < 0.0001), Fpn (F(2,15) = 15.50, *p* = 0.0002), and Fth (F(2,15) = 10.16 *p* = 0.0016) from each group. β-actin was used as a loading control. **E** Representative images of Perls’ blue-stained cortex tissues from the above groups. Magnification is 100/400×. Scale bar is 75/250 μm. Quantification of iron-positive cells per vision field (F(2,15) = 30.22, *p* < 0.0001). **F** The change of Fe^2+^ content (F(2,15) = 12.44, *p* = 0.0007) from each group. **G** Representative gel bands and quantification of 4HNE (F(2,15) = 12.73, *p* = 0.0006) in the injured cortex from each group. β-actin was used as a loading control. **H** Quantitative analysis of GSH content (F(2,15) = 18.78, *p* < 0.0001) from each group. **I** Quantitative analysis of MDA content (F(2,15) = 11.223, *p* = 0.0011) from each group. **J** Representative IF staining to indicate Gpx4-positive cells (red) with nuclei fluorescently labelled with Dapi (blue). Magnification is 400×. Scale bar is 75 μm. Quantification of Gpx4-positive cells per vision field (F(2,15) = 25.33, *p* < 0.0001). Data are shown as mean ± SD (*n* = 6 per group) and analyzed using one-way ANOVA with Bonferroni post hoc test. For all panels, *p < 0.05, **p < 0.01, ***p < 0.001, and ****p < 0.0001. All data are representative of three independent experiments

To explore the role of the Wnt signaling pathway in the pathological process of TBI, Western blotting and immunofluorescence staining were conducted and revealed that TBI resulted in a remarkable decrease of β-catenin (Western botting: t(10) = 11.09, *p* ˂ 0.0001; immunofluorescence staining: t(10) = 5.726, *p* ˂ 0.0001), compared with Sham group (Fig. 5A and B), demonstrating the inhibition of the Wnt signaling pathway post-TBI. However, NaHS treatment effectively inversed the TBI-induced decrease of β-catenin (Fig. 5A and B), indicating that NaHS might activate the Wnt signaling pathway after TBI. Owing to the potent anti-ferroptosis effects of NaHS, it’s reasonable to speculate that activation of the Wnt signaling pathway is possibly involved in the anti-ferroptosis action of NaHS treatment. Then, the intranasal application of recombinant Wnt3a was performed to activate the Wnt signaling pathway. Western blotting results revealed that the increased level of Wnt3a (t(10) = 4.604, *p* = 0.0010) and β-catenin (t(10) = 3.874, *p* = 0.0045) was observed in TBI + Wnt3a group (Fig. 5C), compared with TBI + Vehicle group, confirming the effectiveness of Wnt3a treatment.

Then, iron-associated biomarkers were measured. Compared with TBI + Vehicle group, the expression of Fpn (t(10) = 5.174, *p* = 0.0003) and Fth (t(10) = 4.482, *p* = 0.0013) was significantly increased in the presence of Wnt3a treatment. Furthermore, Wnt3a intervention significantly reduced the increased level of Tfr1 (t(10) = 6.715, *p* ˂ 0.0001) induced by TBI, which indicated that the abnormal iron metabolism was partly restored by Wnt3a treatment (Fig. 5D). In addition, Perl’s blue staining and Fe^2+^ content assay was also performed, and results revealed that Wnt3a treatment significantly decreased iron-positive cells (t(10) = 6.577 *p* ˂ 0.0001) and Fe^2+^ content (t(10) = 4.185, *p* = 0.0024) relative to TBI + Vehicle group (Fig. 5E and F). Importantly, Lip-1 obtained similar results above (Fig. 5E and F). These results demonstrated that TBI-induced abnormal iron metabolism could partly be restored by activating the Wnt signaling pathway.

Furthermore, we explored the effects of Wnt3a or Lip-1 treatment on TBI-induced lipid peroxidation and antioxidant system dysfunction. Western blotting results revealed that Wnt3a intervention significantly reduced the increased level of 4HNE (t(10) = 4.866, *p* = 0.0006) induced by TBI (Fig. 5G). In addition, we also found that treating with Wnt3a increased the GSH content (t(10) = 5.839, *p* ˂ 0.0001) and decreased the MDA content (t(10) = 3.328, *p* = 0.0138) compared with TBI + Vehicle group (Fig. 5H and I). In addition, immunofluorescence staining results showed that the number of Gpx4 positive cells in the TBI + Wnt3a group was significantly higher than that in the TBI + Vehicle group (t(10) = 6.641, *p* ˂ 0.0001) (Fig. 5J). Interestingly, similar results were also obtained by Lip-1 treatment after TBI (Fig. 5G-J), indicating that TBI-induced excessive lipid peroxides and impairments in the antioxidant system were partly restored by Wnt3a or Lip-1 treatment. To conclude, these results revealed that overexpression of the Wnt signaling pathway might lower the sensitivity of cells against ferroptosis post-TBI.

### Wnt3a or Lip-1 Treatment Exerted Protective Effects by Alleviating Ferroptosis and Neuronal Death

**Fig. 6:**
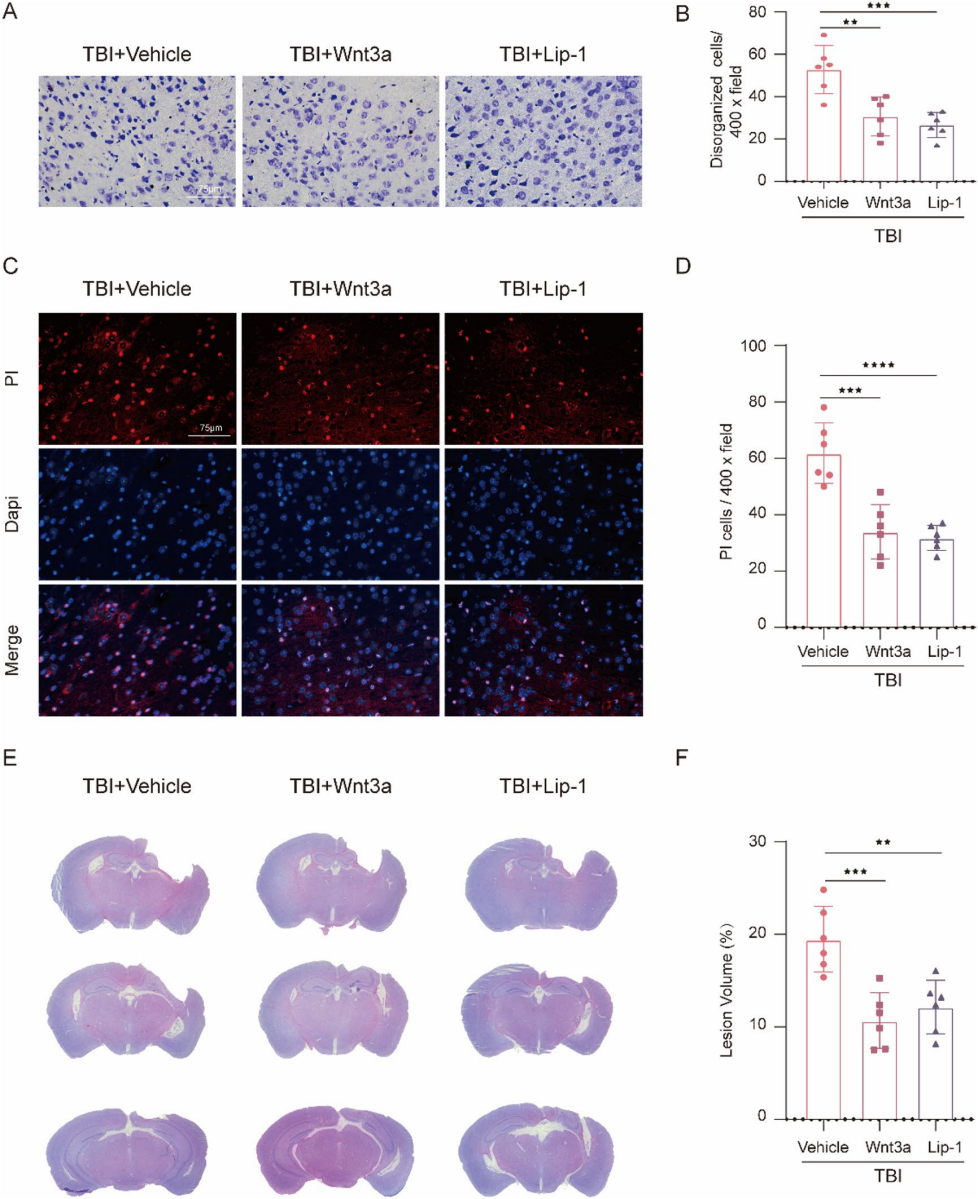
Wnt3a or Lip-1 treatment ameliorated neuronal death and tissue loss after TBI. **A** Representative pictures of Nissl staining in the cortex from the TBI + Vehicle, TBI + Wnt3a, and TBI + Lip-1 group. Magnification is 400×. Scale bar is 75 μm. **B** Quantitative analysis of disorganized cells per vision field (F(2,15) = 14.43, *p* = 0.0003). **C** Representative photomicrographs of double staining with PI (red) and DAPI (blue). Magnification is 400×. Scale bar is 75 μm. **D** Quantification of PI-positive cells per vision field (F(2,15) = 22.22, *p* < 0.0001). **E** Brain sections stained with HE staining. **F** Lesion volume percentage presented as the lesion to contralateral brains (F(2,15) = 13.32, *p* = 0.0005). All data are presented as mean ± SD (*n* = 6) and analyzed using one-way ANOVA with Bonferroni post hoc test. For all panels, **p < 0.01, ***p < 0.001, and ****p < 0.0001. All data are representative of three independent experiments

To further investigate the effects of Wnt3a treatment on TBI-induced neuron damage, Nissl staining was conducted and revealed that Wnt3a treatment significantly reduced the number of disorganized neurons in the injured cortex (t(10) = 4.224, *p* = 0.0022), compared to TBI + Vehicle group (Fig. 6A and B). In addition, we also found that the number of PI-positive cells in the TBI + Wnt3a group was much lower than that in the TBI + Vehicle group (t(10) = 5.545, *p* = 0.0002) (Fig. 6C and D), indicating that Wnt3a treatment effectively inhibits the cell death in injured cortex post-TBI. Moreover, HE staining results revealed that lesion volumes were remarkably smaller after Wnt3a treatment (t(10) = 4.817, *p* = 0.0007) compared to the TBI + vehicle group (Fig. 6E and F). As expected, Lip-1 treatment obtained a similar neuroprotective effect post-TBI (Fig. 6A–F). These results demonstrated that activation of the Wnt signaling pathway could attenuate TBI-induced neuronal loss.

### Wnt3a or Lip-1 Intervention Promoted Neurobehavioral Function Recovery

**Fig. 7:**
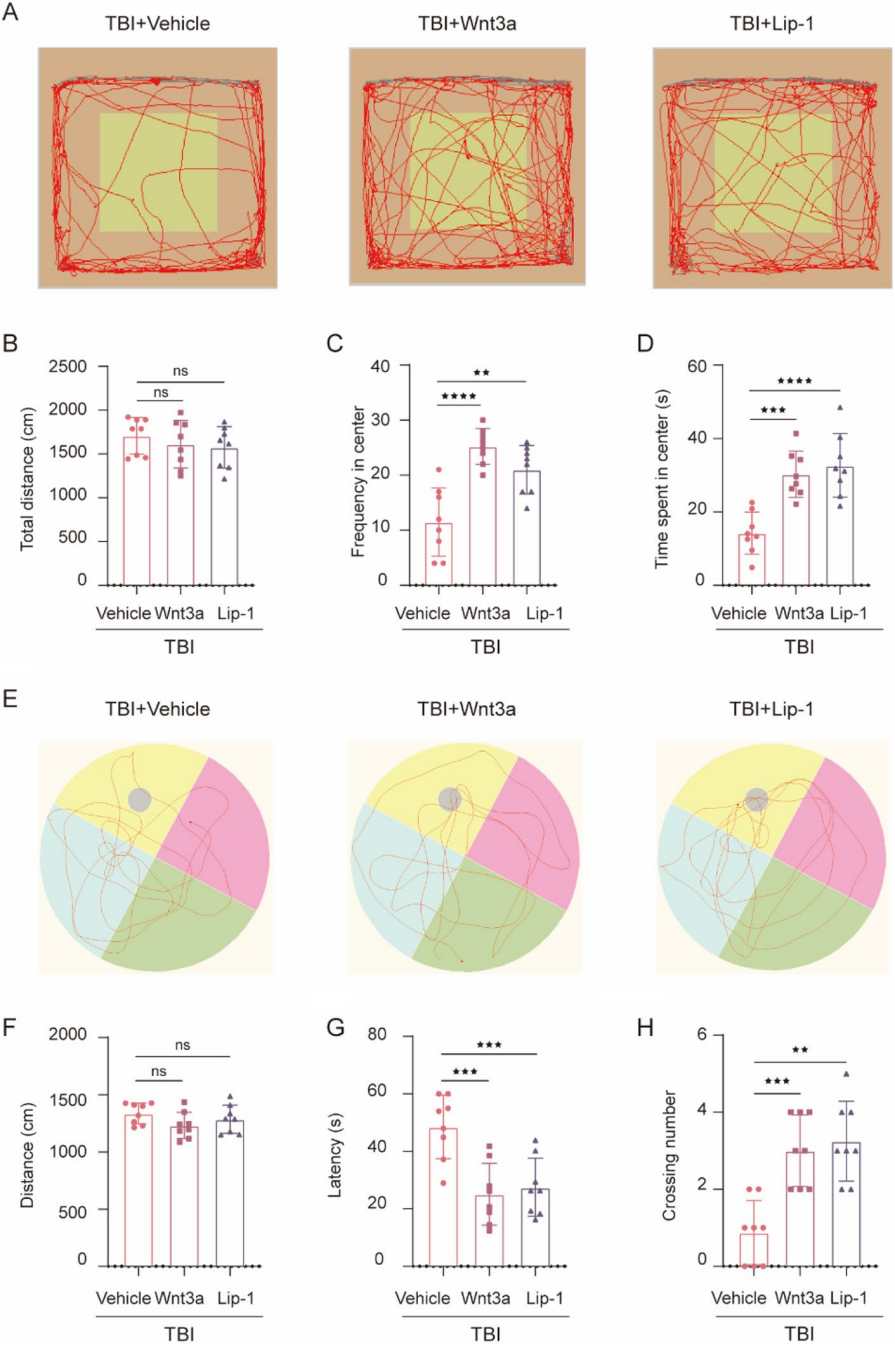
Wnt3a or Lip-1 treatment decreased TBI-induced behavioral deficits in mice. **A**–**D** The effect of Wnt3a or Lip-1 treatment on open field behaviors following TBI. **A** Representative tracks of the open field activity in a mouse from TBI + Vehicle, TBI + Wnt3a, and TBI + Lip-1 group. **B** Total distance traveled moved in the OFT (F(2,21) = 0.6538, *p* = 0.5313). **C** Frequency into the central field by mice during the OFT (F(2,21) = 17.44, *p* < 0.0001). **D** Time spent in the central field by mice in the OFT (F(2,21) = 16.31, *p* < 0.0001). (E–H) The effect of Wnt3a or Lip-1 treatment on the learning and memory ability of mice obtained from all groups. **E** Representative swimming pace of mice from different groups in the MWM test. **F** The traveled distance in the probe trial (F(2,21) = 1.751, *p* = 0.1981). **G** Latency to find the platform in the probe trial (F(2,21) = 11.72, *p* = 0.0004). **H** The number of targets crossing in the probe trial (F(2,21) = 15.57, *p* < 0.0001). All data are presented as mean ± SD (*n* = 8) and analyzed using one-way ANOVA with Bonferroni post hoc test. For all panels: *p < 0.05, **p < 0.01, ***p < 0.001, ****p < 0.0001 and ns means not statistically significant

To explore the effects of Wnt3a or Lip-1 treatment on anxiety behavior, OFT was performed at 8 days following TBI. The mice of the TBI + Wnt3a or TBI + Lip-1 group showed no significant difference in the total distance when compared to the TBI + Vehicle group (F(2,21) = 0.6538, *p* = 0.5313) (Fig. 7B). Interestingly, Wnt3a (t(14) = 5.767, *p* ˂ 0.0001) or Lip-1 (t(14) = 3.985, *p* = 0.0020) treatment significantly inverted the decrease of frequency into the center zone induced by TBI (Fig. 7A and C). And, time spent in the center zone was remarkably increased after Wnt3a (t(14) = 4.561, *p* = 0.0005) or Lip-1 (t(14) = 5.256, *p* ˂ 0.0001) treatment following TBI (Fig. 7D), indicating that Wnt3a or Lip-1 intervention both attenuated the anxiety level after TBI. Then we detected the long-term learning and memory function from 10 to 14 days post-TBI. Data demonstrated that no statistical difference in swimming distance was found among the above groups (F(2,21) = 1.751, *p* = 0.1981) (Fig. 7F). Noteworthy, compared to the TBI + Vehicle group, an increase in crossing number through the platform area (t(14) = 4.543, *p* = 0.0005) and a decrease in escape latency (t(14) = 4.401, *p* = 0.0007) were detected in TBI + Wnt3a group (Fig. 7E, G and H), suggesting that Wnt3a treatment might be beneficial for the recovery of spatial memory in TBI mice. Lip-1 intervention also obtained similar results with Wnt3a treatment (Fig. 7E–H). To summarize, both Wnt3a and Lip-1 treated mice showed less anxiety and spatial memory deficits after TBI.

### Activation of WNT Signaling Pathway Contributed to the Anti-ferroptosis Effects and Neurological Recovery of NaHS Treatment After TBI

**Fig. 8:**
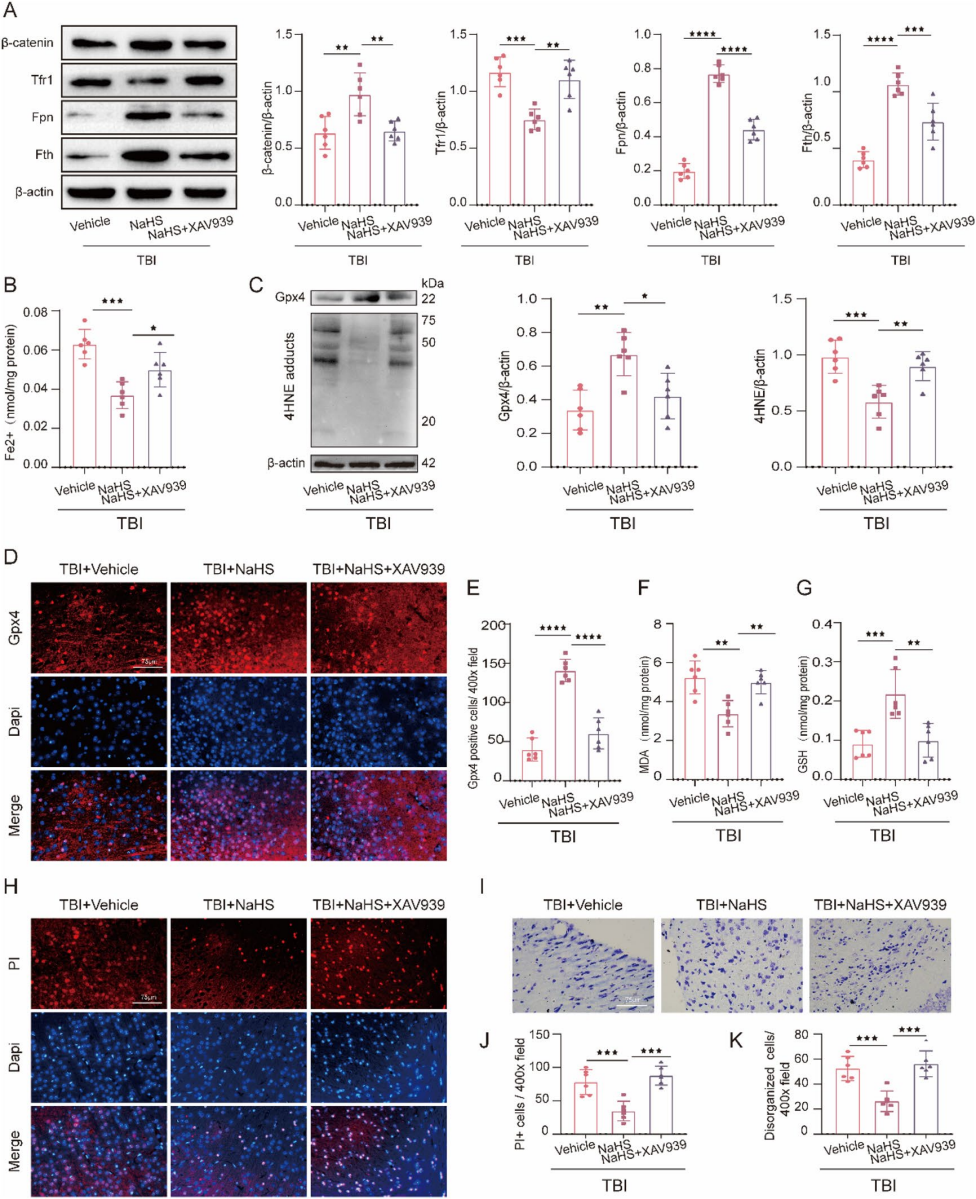
NaHS prevented TBI-induced ferroptosis and neuronal damage at least partly through Wnt signaling pathway. **A** Representative Immunoblot and quantification of β-catenin (F(2,15) = 10.31, *p* = 0.0015), Tfr1 (F(2,15) = 16.95, *p* < 0.0001), Fpn (F(2,15) = 181.1, *p* < 0.0001), Fth (F(2,15) = 48.68, *p* < 0.0001) and β-actin in injured cortex obtained from TBI + Vehicle, TBI + NaHS and TBI + NaHS + XAV939 group. β-actin was used as a loading control. **B** The change of Fe^2+^ content (F(2,15) = 16.68, *p* = 0.0002, G) from each group. **C** Representative gel bands and quantification of Gpx4 (F(2,15) = 11.02, *p* = 0.0011) and 4HNE (F(2,15) = 13.49, *p* = 0.0004) from above groups. **D** Representative images of immunofluorescent staining for Gpx4 (red) and Dapi (blue). Magnification is 400×. Scale bar is 75 μm. **E** Quantification of Gpx4 positive cells per vision field (F(2,15) = 62.83, *p* < 0.0001). **F** The change of MDA content (F(2,15) = 12.07, *p* = 0.0008) from each group. **G** The change of GSH (F(2,15) = 13.18, *p* = 0.0005) content from each group. **H** Representative photomicrographs of double staining with PI (red) and DAPI (blue). Magnification is 400×. Scale bar is 75 μm. **I** Representative Nissl staining sections from the above groups. Magnification is 400×. Scale bar is 75 μm. **J** Quantification of PI-positive cells per vision field (F(2,15) = 18.62, *p* < 0.0001). **K** Quantification of disorganized cells per vision field (F(2,15) = 17.78, *p* < 0.0001). All data are presented as mean ± SD (*n* = 6) and analyzed using one-way ANOVA with Bonferroni post hoc test. For all panels: *p < 0.05, **p < 0.01, ***p < 0.001, and ***p < 0.0001. All data are representative of three independent experiments

Many studies have verified XAV939 as a new small molecule inhibitor of the Wnt/β-catenin pathway, which binds and inhibits tankyrase, thereby leading to increased β-catenin destruction (Jang et al. 2019; Katoh 2018). Notably, the aggravation effect of XAV939 on neurological damage after brain injury has been reported in previous literature (Fei et al. 2020; Guo et al. 2023). To ascertain whether the Wnt signaling pathway was involved in the anti-ferroptosis effects of NaHS treatment after TBI, we conducted the administration of XAV939 to inhibit Wnt signaling pathway post-TBI. Western blotting results revealed that XAV939 led to a remarkable downregulation of β-catenin in the cortex (t(10) = 3.894, *p* = 0.0043) (Fig. 8A), confirming the high efficiency of XAV939.

Firstly, Western blotting was conducted to detect the changes in iron-associated markers (Tfr1, Fpn, and Fth). The results showed that XAV939 treatment increased the expression of Tfr1 (t(10) = 4.561, *p* = 0.0011) and decreased the expression of Fpn (t(10) = 10.87, *p* ˂ 0.0001) and Fth (t(10) = 4.882, *p* = 0.0006), compared with TBI + NaHS group (Fig. 8A). In addition, we also found that downregulation of Fe^2+^ caused by TBI + NaHS was markedly reversed after XAV939 treatment (t(10) = 2.888, *p* = 0.0338) (Fig. 8B). These results revealed that XAV939 could aggravate the iron metabolism impairments post-TBI.

Then, we explored the effects of XAV939 on markers associated with lipid peroxidation and the antioxidant system. Western blotting results showed that XAV939 intervention reversed TBI + NaHS-induced increase of Gpx4 (t(10) = 3.384, *p* = 0.0123) and decrease of 4HNE (t(10) = 3.894, *p* = 0.0006) (Fig. 8C). And, immunofluorescence staining results also showed that the number of Gpx4 positive cells in TBI + NaHS + XAV939 group was significantly lower than that in TBI + NaHS group (t(10) = 8.436, *p* ˂ 0.0001) (Fig. 8D and E), which supported the results of Western blotting. Moreover, we measured the GSH and MDA content and found that XAV939 significantly inverted the increase of GSH (t(10) = 4.279, *p* = 0.0020) and decrease of MDA (t(10) = 3.932, *p* = 0.0040) caused by NaHS after TBI (Fig. 8F and G). These results revealed that inhibition of the Wnt signaling pathway might deteriorate TBI-induced lipid peroxidation and antioxidant system disorders.

Finally, we investigated the effect of XAV939 on TBI-induced neuronal damage. PI-staining results showed that XAV939 effectively increased the number of PI-positive cells compared to the TBI + NaHS group (t(10) = 5.729, *p* ˂ 0.0001) (Fig. 8H, J), indicating the severity of cell death was aggravated by inhibition of the Wnt signaling pathway. In addition, the results from Nissl staining demonstrated that XAV939 increased the seriousness of neuron damage as evidenced by the increased number of damaged neurons, shrunken cytoplasm, and triangular nucleus, compared with TBI + NaHS group (t(10) = 5.468, *p* = 0.0002) (Fig. 8I, K). Overall, these results provide substantial evidence to support that NaHS intervention negatively regulates ferroptosis, at least partially via activating the Wnt signaling pathway.

## Discussion

**Fig.9:**
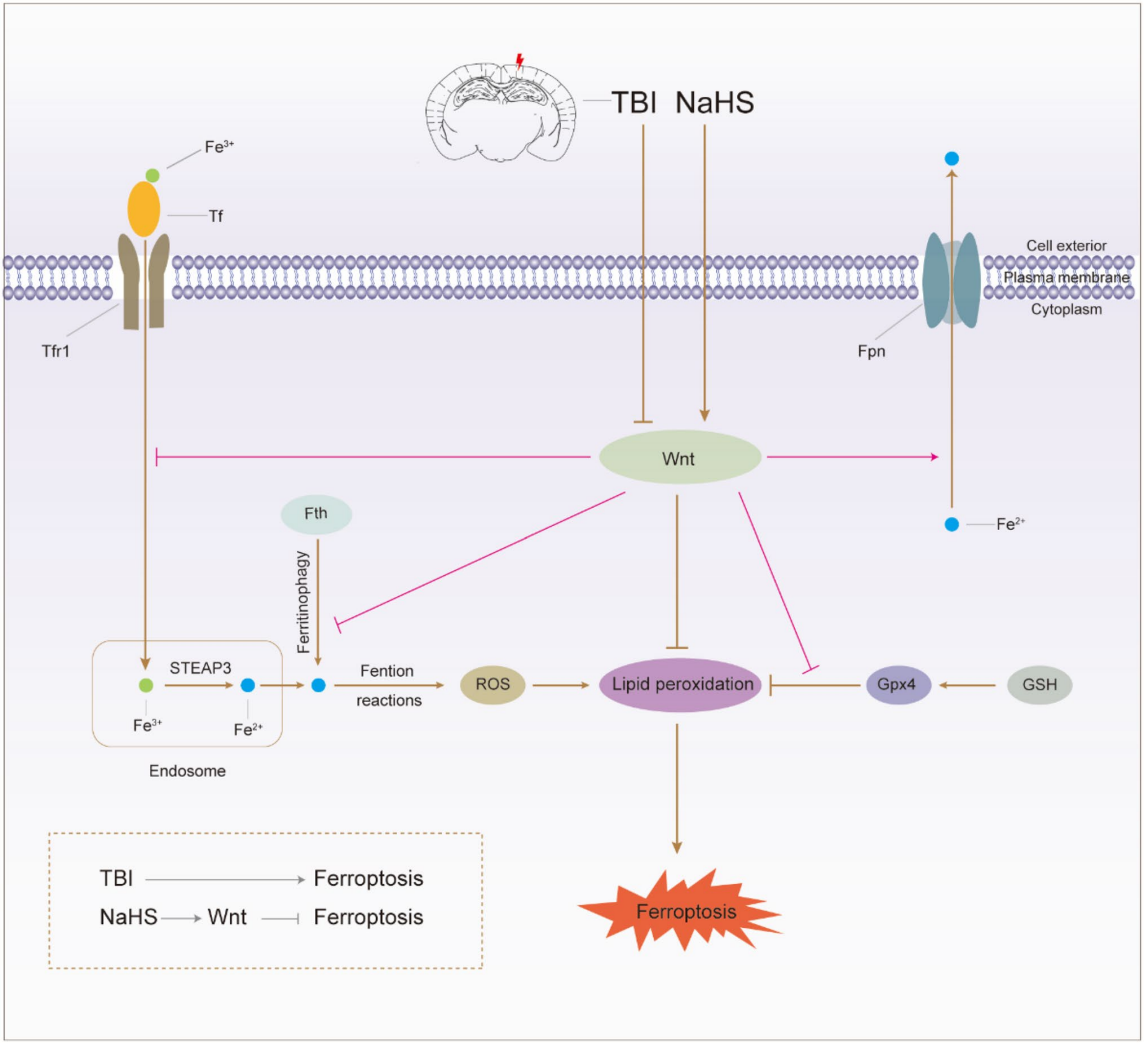
The schematic representation of neuroprotective effects of NaHS treatment against TBI-induced ferroptosis. TBI induces ferroptosis-related changes characterized by iron accumulation, decreased Gpx4, and increased lipid peroxidation at the chronic phase after TBI. However, NaHS treatment reduces the susceptibility to TBI-induced ferroptosis, at least partly by activating the Wnt signaling pathway

The critical role of H_2_S in inhibiting ferroptosis and promoting function recovery against TBI has been reported in many previous studies (Chen et al. 2022; Kimura et al. 2006; Sun et al. 2021). However, the underlying mechanism remains unclear. This study explored whether the Wnt/β-catenin pathway was involved in the anti-ferroptosis effects of H_2_S post-TBI. We first examined the effects of H_2_S on TBI-induced ferroptosis through Western blotting, immunofluorescent staining, and Fe^2+^, MDA, and GSH content assay. As expected, the results revealed that H_2_S remarkably rescued cells from ferroptosis at the chronic stage following TBI. Then, we found that the Wnt signaling pathway was significantly inhibited after TBI, and activating the Wnt signaling pathway could remarkably decrease TBI-induced ferroptosis. Finally, further mechanistic investigations revealed that inhibition of the Wnt signaling pathway could partly abolish the anti-ferroptosis and neuroprotective effects induced by H_2_S treatment after TBI. To sum up, these results provided potent evidence that the anti-ferroptosis effects of H_2_S against TBI were established at least in part via the Wnt signaling pathway, as shown schematically in Fig. 9.

The pathophysiology of TBI includes primary injury and secondary injury. The primary injury caused by external mechanical force in the impact site of the brain is transient and irreversible, resulting in tissue distortion, axonal shearing, vascular damage, and extensive cell loss. However, due to the limitation of treatment targeting the primary injury, exploring the molecular mechanisms in the pathological process of the secondary injury has been a consensus among researchers (Jassam et al. 2017). The secondary injury occurs within a few minutes after the primary injury. It involves a series of multifactorial events, including glutamatergic excitotoxicity, increased vascular permeability, mitochondrial dysfunction, and neuroinflammation, which may last for several months and ultimately results in different types of cell death, such as apoptosis, autophagy, and pyroptosis within the surrounding area. In addition, inhibiting various cascades of cell death during secondary injury has become an effective therapeutic strategy to facilitate neurological recovery after TBI (Lin et al. 2023). In recent years, growing evidence shows that ferroptosis, a novel form of cell death, is highly complicated in the pathological development of secondary brain injury post-TBI. Moreover, it has been proved that inhibiting ferroptosis can significantly reduce cognitive defects and motor dysfunction in the mouse TBI model (Geng et al. 2021; Xie et al. 2019). For example, Xie et al. reported that typical characteristics of ferroptosis, including abnormal iron metabolism, overexpression of ferroptosis‐related genes, and inactivation of GPx, were detected in the ipsilateral cortex at 3 days post-TBI (Xie et al. 2019). Furthermore, several studies have revealed that melatonin or mesenchymal stromal cells (MSCs) treatment could reduce neuronal degeneration and alleviate persistent cognitive impairments after TBI, possibly by inhibiting ferroptosis (Rui et al. 2021; Wang et al. 2022a, b, c, d).

Ferroptosis, a new cell death type, is driven by iron overload, impaired glutathione metabolism, and massive lipid peroxidation, which is morphologically, biochemically, and genetically distinct from other classical cell death pathways such as apoptosis (Jiang et al. 2021). As is evident from the name itself, iron plays a vital role in ferroptosis. Due to the critical role of iron in multiple physiological processes, such as mitochondrial respiration, DNA synthesis, or cell signaling, it’s not a surprise that iron metabolism is under exquisite control mainly through the regulation of iron importing, storage, and exporting (Tang et al. 2021). As a critical regulator of iron importing, transferrin receptor-1 (Tfr1) is responsible for increasing iron intake. As for iron storage, Ferritin, which consists of ferritin light chain (Ftl) and ferritin heavy chain (Fth), can store 70%-80% of newly imported iron (Chen et al. 2021a, b). Notably, the function of Fth is to catalyze the oxidation of Fe^2+^ to Fe^3+^, avoiding excess production of free Fe^2+^ in the cytoplasm. In addition, Ferroportin (Fpn), the only known iron exporter protein, is responsible for extruding Fe^2+^ into the extracellular space, thereby reducing intracellular Fe^2+^ content and maintaining cellular iron homeostasis. Conceivably, the regulation of essential proteins in iron metabolism is considered to alter the sensitivity of cells to ferroptosis (Tang et al. 2021). For example, genetically silencing Tfr1 can effectively inhibit the cellular iron import, rendering cells more resistant to ferroptosis (Gao et al. 2015). Otherwise, the depletion of Fth remarkably facilitated the susceptibility to ferroptosis via upregulation of iron level and lipid ROS (Fang et al. 2020; Rui et al. 2021). Consistent with previous literature (Chen et al. 2021a, b; Gao et al. 2021), we found that TBI induced the upregulation of Fe^2+^ and Tfr1, as well as downregulation of Fpn in the injured cortex (Fig. 2A), indicating that the increase of iron importing and decrease of iron exporting induced by TBI work together to cause iron overload, thereby promoting the progression of ferroptosis. Surprisingly, the results of our study also revealed that Fth, an anti-ferroptosis protein, was increased following TBI. However, increased Fth induced by TBI could make cells more resistant to ferroptosis. Regarding this inconsistent observation, we speculated that the overexpression of Fth might be a self-compensatory response against TBI. Moreover, we found that H_2_S treatment not only reversed the TBI-induced increase of Tfr1 and decrease of Fpn but also promoted the increase of Fth induced by TBI (Fig. 2A). To sum up, the changes of Tfr1, Fth, and Fpn post-H_2_S treatment all demonstrated the TBI-induced abnormal iron metabolism might be partly restored by H_2_S.

Lipid peroxidation, the main driver of ferroptosis, refers to the process in which oxidants (e.g., free radical substances or non-free radical substances) attack the polyunsaturated fatty acids of cell membranes, resulting in rapid and unrepairable oxidative damage of membranes (Chen et al. 2021a, b). It should be noted that the level of 4-Hydroxynonenal (4HNE) or MDA, decomposing products of lipid hydroperoxides, could be considered to reflect the severity of lipid peroxidation (Conrad and Pratt 2019). In addition, the lipid peroxidation reactions would likely be terminated by the cyst(e)ine–GSH–Gpx4 antioxidant system (Chen et al. 2021a, b; Miess et al. 2018), and the impaired cyst(e)ine–GSH–Gpx4 antioxidant system is another vital feature of ferroptosis. As a critical phospholipid hydroperoxide, glutathione peroxidase 4 (Gpx4) can reduce lipid peroxides to the corresponding alcohols, protecting cells against lethal lipid ROS attack, which is considered to be the ferroptosis gatekeeper and plays a crucial role in preventing membrane lipid peroxidation. Notably, maintaining the proper function of Gpx4 requires the availability of GSH, which can provide two electrons necessary for Gpx4 activity and is the most abundant cellular reductant (Maiorino et al. 2018). In this study, we found increased expression of MDA and 4HNE, as well as decreased expression of GSH and Gpx4 in the impaired cortex after TBI (Fig. 2G–M), which was consistent with previous observations that TBI resulted in lipid peroxidation and inferior Gpx4 antioxidant system (Chen et al. 2021a, b; Wang et al. 2022a, b, c, d; Xie et al. 2019). After that, we also found that H_2_S treatment significantly reduced those alterations above (Fig. 2G–M), providing robust evidence to support the anti-ferroptosis action of H_2_S against TBI.

The above results in this research have demonstrated the critical role of H_2_S in desensitizing cells from ferroptosis after TBI, consistent with many previous studies (Li et al. 2022; Sun et al. 2021; Wang et al. 2022a, b, c, d). It should be noted that ferroptosis is one of the cell death types induced by TBI (Gao et al. 2023). As shown in Figs. 3 and 6, we found that H_2_S or Lip-1 treatment decreased only 40–50% of PI-positive cells, indicating that almost 50–60% of TBI-induced cell death was driven by other cell death forms such as apoptosis, autophagy, pyroptosis, and necrosis. Thus, the combination of multiple inhibitors of cell death has become a potential therapeutic strategy for treating TBI (Hu et al. 2022a, b). In addition, results from Figs. 4 and 7 revealed that TBI-induced spatial memory deficits and increased anxiety levels were almost restored to normal condition by H_2_S or Lip-1 treatment, which raised a problem about why the survival of only partial cells could almost maintain cognitive function. About this dispute, we explained that cognition is regarded as the most complex function of the brain. Cognitive function is the result of the interaction of multiple networks in the whole brain area, which is based on all three levels of the brain tissue: molecular/cell level, local circuit level, and large-scale network level, not just the cell death level in the injured cortex (Birle et al. 2021). Therefore, we speculated that the restoration of cognitive function might be attributed to the comprehensive neuroprotective effects of H_2_S post-TBI in both the damaged cortex and other brain regions, such as the hippocampus, prefrontal cortex, and corpus striatum.

Then, we investigated the specific molecular mechanisms by which H_2_S represses the process of ferroptosis post-TBI. As a highly evolutionary conserved signal transduction cascade, the Wnt/β-catenin signaling pathway has been recently reported to be highly implicated in the execution of ferroptosis, and targeting the Wnt signaling pathway could exacerbate melanoma cell ferroptosis (Wang et al. 2022a, b, c, d). In addition, several studies have shown that the multiple physiological effects of H_2_S against many diseases mainly depend on the Wnt signaling pathway (Bhattacherjee et al. 2022; Zhang and Ye 2019). Given the high conservatism of the Wnt signaling pathway, the anti-ferroptosis effect of H_2_S treatment following TBI may be partly mediated through the Wnt signaling pathway. To verify this hypothesis, we performed intranasally administration of Wnt3a to overexpress the Wnt signaling pathway following TBI and explore the ferroptosis-related changes (Matei et al. 2018; Zhang et al. 2018). Intriguingly, the results of our study revealed that Wnt3a treatment significantly reduced TBI-induced ferroptosis (Fig. 5C–J), as evidenced by decreasing iron overload, mitigating lipid peroxidation, as well as increasing the activity of Gpx4, which obtained the similar results from Lip-1, providing the first evidence of anti-ferroptosis action of Wnt signaling pathway following TBI. Then, we conducted the administration of XAV939 (a Wnt signaling pathway inhibitor) along with the H_2_S treatment after TBI (Song et al. 2021), aiming to explore whether the inactivation of the Wnt signaling pathway could reverse the alleviation of ferroptosis induced by H_2_S. Previous studies have demonstrated that XAV939 treatment could aggravate neurological deficits after brain injury, as evidenced by increased brain edema and BBB disruption (Zhao et al. 2020). However, the effect of XAV939 on TBI-induced ferroptosis remains to be unclear. The results in this study revealed that XAV939 treatment mostly abolished the neuroprotective effects of H_2_S against TBI-induced ferroptosis and neuronal damage (Fig. 8), as demonstrated by increased levels of Fe^2+^, Tfr1, 4HNE, and MDA, as well as decreased levels of GSH, Gpx4, Fpn, and Fth. To sum up, this study ultimately reveals that H_2_S intervention significantly inhibits TBI-induced ferroptosis, at least in part, via the Wnt/β-catenin signaling pathway. These underlying mechanisms might provide a new theoretical basis for the clinical translational application of H_2_S for treating TBI, thereby promoting the neurological function recovery of TBI patients.

## Conclusion

Although previous studies have reported that H_2_S could exert neuroprotective effects by reducing ferroptosis post-TBI (Kimura et al. 2006; Sun et al. 2021), this study first demonstrated that the Wnt/β-catenin pathway might be the critical mechanism of the anti-ferroptosis effects of H_2_S against TBI. These data provided a substantial theoretical basis for the clinical application of H_2_S therapy in the treatment of TBI. Despite the focus of this study being on TBI, this highly translatable intervention could also be applied to other neurological disorders. However, there are still several limitations in our study. Firstly, owing to the multiple biologic functions of H_2_S, we cannot rule out the possibility that the beneficial effects of H_2_S in facilitating brain function recovery were also mediated by other mechanisms apart from inhibiting ferroptosis. Therefore, the multiple interaction mechanisms among ferroptosis and other effects of H_2_S, such as anti-inflammation and anti-apoptosis, need to be explored further. Secondly, it is necessary to investigate the optimal dose and timing of H_2_S intervention after TBI, as well as the distribution and metabolism of H_2_S in future research. Furthermore, since only male mice were involved in this study, follow-up studies in female mice are required, decreasing the experimental bias caused by gender factors.

### Supplementary Information

Below is the link to the electronic supplementary material.

Supplementary file1 (PDF 10694 KB)

## Data Availability

All data generated or analyzed in this study are available from the corresponding author on reasonable request.

## Abbreviations

TBI: Traumatic brain injury
H_2_S: Hydrogen sulfide
LPS: Lipopolysaccharide
Gpx4: Glutathione peroxidase 4
Lip-1: Liproxstatin-1
NaHS: Sodium hydrosulfide
mTBI: Mild TBI
AD: Alzheimer disease
PD: Parkinson’s disease
BBB: Blood–brain barrier
T1DM: Type 1 diabetes mellitus
PM2.5: Fine particulate matter, with less than 2.5 μm in aerodynamic diameter
ROS: Reactive oxygen species
GC: Gastric cancer
DCM: Diabetic cardiomyopathy
CCI: Controlled cortical impact
DMSO: Dimethyl sulfoxide
PEG 300: Polyethylene glycol 300
ddH_2_O: Double distilled water
BSA: Bovine serum albumin
OFT: Open field test
MWM: Morris water maze
WB: Western blotting
IF: Immunofluorescence
HE: Hematoxylin–eosin
PI: Propidium iodide
MDA: Malondialdehyde
PMSF: Phenylmethanesulfonyl fluoride
BCA: Bicinchoninic acid assay
PVDF: Polyvinylidene fluoride
PFA: Paraformaldehyde
PBS: Phosphate-buffered saline
PBST: Phosphate-buffered saline plus Tween-20
DAB: 3,3‐Diaminobenzidine
TBA: Thiobarbituric acid
Total GS: Total glutathione
GSH: Reduced glutathione
GSSG: Oxidized glutathione
Fe^2+^: Ferrous ion
MSCs: Mesenchymal stromal cells
Tfr1: Transferrin receptor-1
Ftl: Ferritin light chain
Fth: Ferritin heavy chain
Fpn: Ferroportin
4HNE: 4-Hydroxynonenal
