# Supplementary material for: Neuroprotective Effect of Hydrogen Sulfide Subchronic Treatment Against TBI-Induced Ferroptosis and Cognitive Deficits Mediated Through Wnt Signaling Pathway
Source: Cell Mol Neurobiol. 2023 Aug 25;43(8):4117–40. doi: 10.1007/s10571-023-01399-5 (PMC10661805; doi:10.1007/s10571-023-01399-5)

Original WB images of Figure 2

Tfr1

Figure 2A

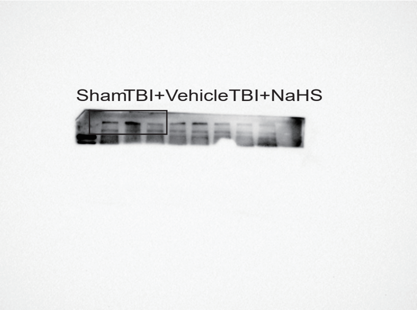

Fpn

Figure 2A

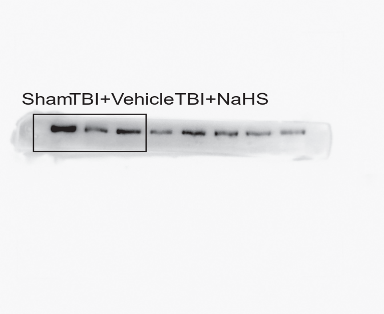

Fth

Figure 2A

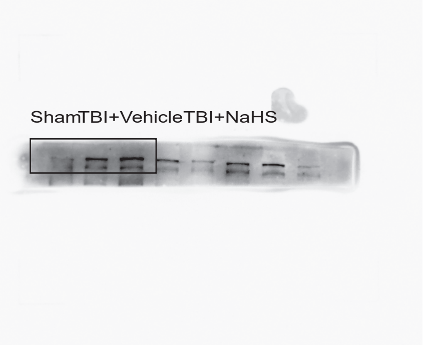

Actin

Figure 2A

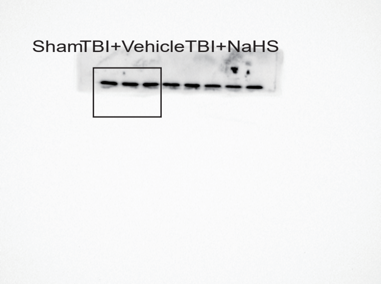

Gpx4

Figure 2G

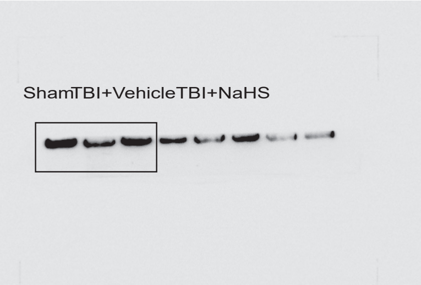

4HNE

Figure 2G

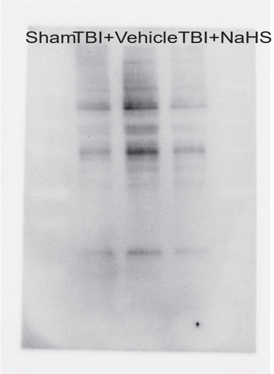

Actin

Figure 2G

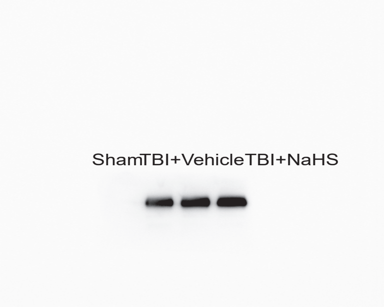

Original WB images of Figure 5

β-catenin

Figure 5A

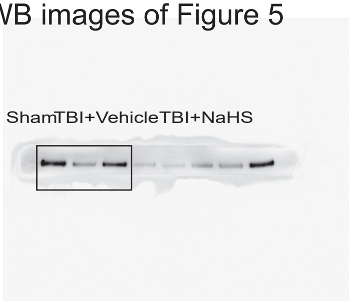

Actin

Figure 5A

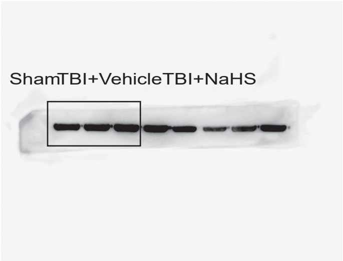

Wnt3a

Figure 5C

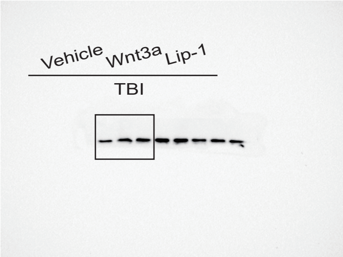

β-catenin

Figure 5C

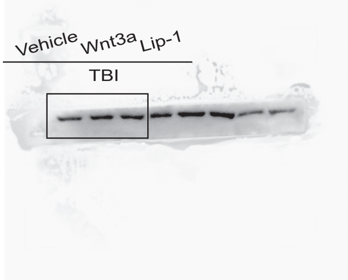

Tfr1

Figure 5C

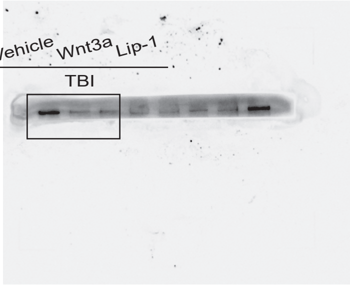

Fth

Figure 5C

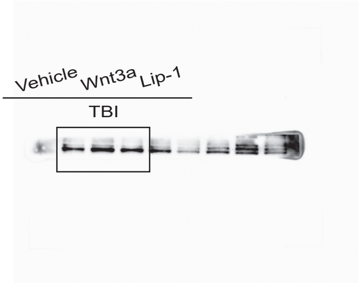

Fpn

Figure 5C

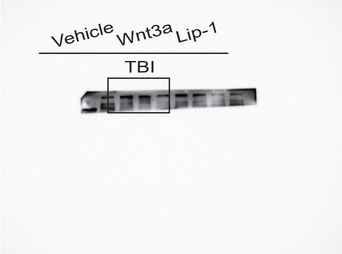

Actin

Figure 5C

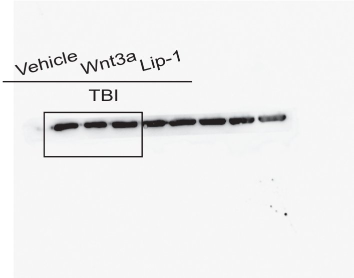

4HNE

Figure 5G

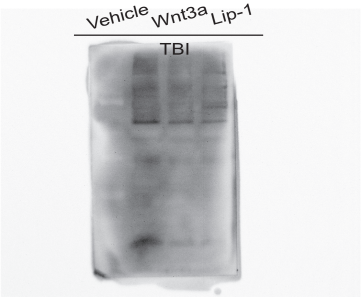

Actin

Figure 5G

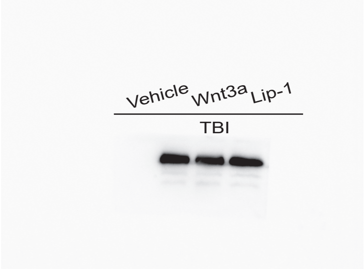

Original WB images of Figure 8

Tfr1
Figure 8A

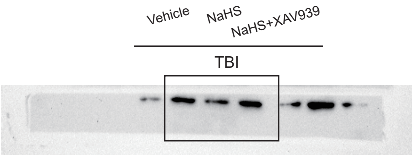

β-catenin
Figure 8A

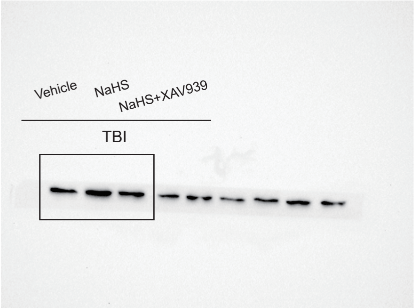

Fpn
Figure 8A

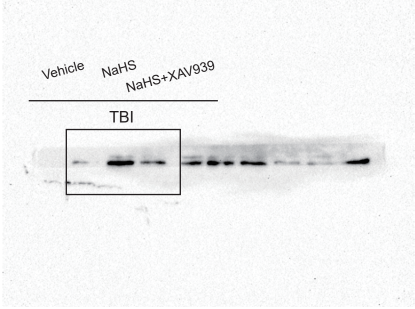

Fth
Figure 8A

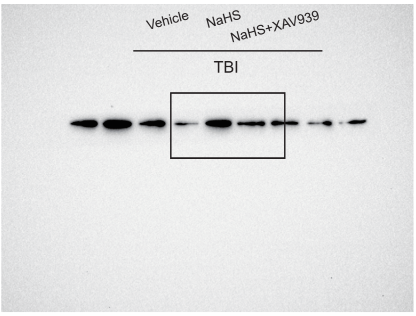

Actin
Figure 8A

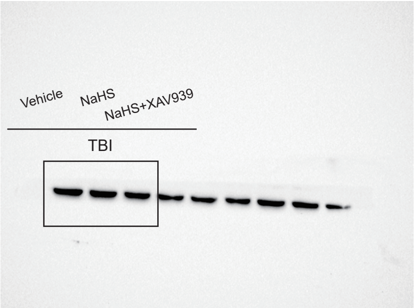

4HNE
Figure 8C

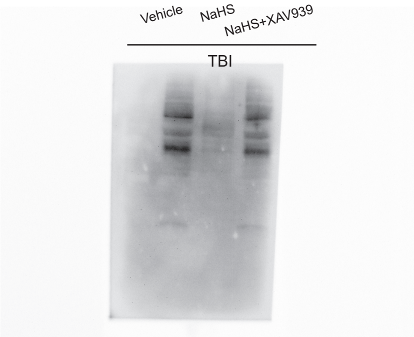

Gpx4
Figure 8C

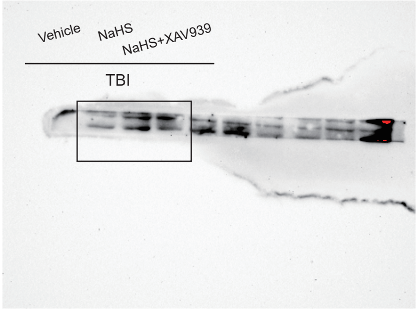

Actin
Figure 8C

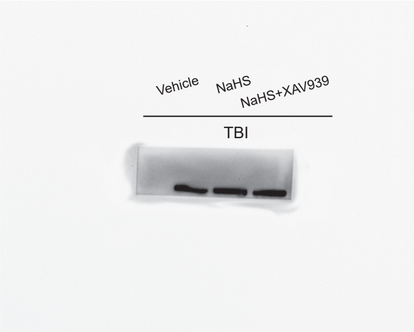

Supplement: Supplementary file 1 — Supplementary file1 (PDF 10694 KB) [file 10571_2023_1399_MOESM1_ESM.pdf]
